# Low-Cost Probabilistic 3D Denoising with Applications for Ultra-Low-Radiation Computed Tomography

**DOI:** 10.3390/jimaging8060156

**Published:** 2022-05-31

**Authors:** Illia Horenko, Lukáš Pospíšil, Edoardo Vecchi, Steffen Albrecht, Alexander Gerber, Beate Rehbock, Albrecht Stroh, Susanne Gerber

**Affiliations:** 1 Faculty of Mathematics, Technical University of Kaiserslautern, 67663 Kaiserslautern, Germany; 2 Department of Mathematics, VSB Ostrava, Ludvika Podeste 1875/17, 708 33 Ostrava, Czech Republic; lukas.pospisil@vsb.cz; 3 Institute of Computing, Faculty of Informatics, Universitá della Svizzera Italiana (USI), 6962 Viganello, Switzerland; edoardo.vecchi@usi.ch; 4 Institute of Physiology, University Medical Center of the Johannes Gutenberg—University Mainz, 55128 Mainz, Germany; s.albrecht@uni-mainz.de; 5 Institute of Occupational Medicine, Faculty of Medicine, GU Frankfurt, 60590 Frankfurt am Main, Germany; dr.a.gerber@gmx.de; 6 Lung Radiology Center Berlin, 10627 Berlin, Germany; dr.b.rehbock@gmx.de; 7 Institute of Pathophysiology, University Medical Center of the Johannes Gutenberg—University Mainz, 55128 Mainz, Germany; albrecht.stroh@unimedizin-mainz.de; 8 Institute for Human Genetics, University Medical Center of the Johannes Gutenberg—University Mainz, 55128 Mainz, Germany

**Keywords:** denoising, nonparametric methods, Mumford–Shah formalism, LAR reduction

## Abstract

We propose a pipeline for synthetic generation of personalized Computer Tomography (CT) images, with a radiation exposure evaluation and a lifetime attributable risk (LAR) assessment. We perform a patient-specific performance evaluation for a broad range of denoising algorithms (including the most popular deep learning denoising approaches, wavelets-based methods, methods based on Mumford–Shah denoising, etc.), focusing both on accessing the capability to reduce the patient-specific CT-induced LAR and on computational cost scalability. We introduce a parallel Probabilistic Mumford–Shah denoising model (PMS) and show that it markedly-outperforms the compared common denoising methods in denoising quality and cost scaling. In particular, we show that it allows an approximately 22-fold robust patient-specific LAR reduction for infants and a 10-fold LAR reduction for adults. Using a normal laptop, the proposed algorithm for PMS allows cheap and robust (with a multiscale structural similarity index >90%) denoising of very large 2D videos and 3D images (with over $10^{7}$ voxels) that are subject to ultra-strong noise (Gaussian and non-Gaussian) for signal-to-noise ratios far below 1.0. The code is provided for open access.

## 1. Introduction

Computed tomography (CT) is one of the most frequently used medical imaging techniques, with over 100 million CT scans performed yearly worldwide [1]. An additional increase in the total number of CT examinations was observed in the recent COVID-19 epidemics [2,3]. However, distinguishing subtle CT image features relevant for diagnostic purposes typically requires significant radiation exposure, and thus increases the patient’s radiation-imposed lifetime attributable risk (LAR). This, in turn, leads to an additional chance of developing a radiation-exposure attributable cancer type [1].

The quantification of LAR is a complex challenge and requires modeling the multifactorial interplay of DNA damage and repair mechanisms, as well as incorporating random/stochastic effects that accumulate in the low-radiation regime. In silico simulations and analytical estimates for net effects of such stochastic radiation-triggered reactions imply a linear model for the dependence of LAR on the accumulated radiation exposure [4,5,6,7], with linear model coefficients being dependent on the patient’s age and sex, as well as on the particular type of the CT. Despite some controversy regarding the possible existence of low-radiation thresholds in the LAR models suggested by some studies [8], the Linear No-Threshold models (LNT) are currently recommended for LAR assessment by the committee for Biologic Effects of Ionizing Radiation (BEIR VII) of the National Academy of Sciences of the USA [5] and by the World Health Organization [1]. Several recent epidemiological and methodological studies support the statement that a safe radiation dose does not exist [9,10,11,12] and that the LAR of CT is exceptionally high for infants and children [10,11,12]. The approximately 14 million pediatric CT scans of head, abdomen, pelvis, chest, or spine performed each year worldwide [1,10] would therefore lead to approximately 12,000 fatal cases of cancer, of which 4,800 are attributable to the USA alone.

The prognosis that the reduction of the highest 25% of doses to the median could prevent 43% of these cancers [10] naturally suggests the increased use of low- and ultra-low-radiation CT (radiation exposures down to 0.5 mGy). However, a reduction of radiation exposure results in increased image noise and thus necessitates the application of reliable image denoising and feature extraction tools. Facilitated by the rapid development of emergent machine learning (ML) and deep learning (DL) algorithms, research on the boundary between medical radiology and informatics has been attracting an increasing amount of attention over the past years [13]. The currently available CT image denoising tools can be roughly subdivided into unsupervised and supervised methods. The unsupervised approaches search for a hidden pattern without prior learning, whereas the supervised techniques aim to identify features previously learned from the training data. Unsupervised methods do not require previous training, allow high-speed computations, and belong to the most frequently-used image denoising instruments [14,15]. They include methods based on local averaging of the data (such as Gaussian, weighted Gaussian, bilateral, and mean average filtering) [14,16,17,18] and spectral methods (such as Fourier-, wavelet- and PCA-denoising) [15,19,20,21,22]. Recent years have also seen the active development of very successful CT denoising approaches based on semi-supervised ML ideas (for example, methods based on generative adversarial networks) [23,24] and deep-learning algorithms for denoising and image segmentation [13,25,26,27]. The deep learning methods have been shown to be very successful for denoising and the current convention states that DL performs much better than traditional unsupervised regularized denoising algorithms.

However, recent evidence in the literature indicates that ML and DL tools can struggle when dealing with the denoising of real images, either due to the lack of an adequate training samples, inaccurate priors, concept drift, or the increasing complexity (and computational cost) of the required network [28,29].

This is particularly true in medical imaging, where the approaches based on ML can sometimes lack accuracy [30], while DL tools tend to rely too heavily on labeled datasets and on sufficiently large training sets [29,31,32,33]. The size of the training set, as well as the accuracy of prior data used in training, play a very central role also in the denoising and segmentation of CT images, where the number of instances in the training set *T* is significantly smaller than the feature space dimension *D*, corresponding to the number of voxels. A problem characterized by D≫T pertains to the so-called “small-data learning challenge” [34,35,36,37,38], and represents a scenario in which ML and DL approaches are prone to quickly overfit the small training set (which in addition often also contains missig data or incorrectly labeled data) and to achieve an unsatisfactory performance on the validation set [39,40,41,42,43]. To tackle this issue, several alternative approaches have been proposed [44,45], with transfer learning representing one of the most powerful alternatives [46]. Even the latter approach presents, however, some limitations that are particularly relevant in the denoising of CT images; due to the individual variation of small-scale anatomical features and of CT operation regimes, the structural similarity assumption between the source domain and the target domain is usually not fulfilled, while the amount and type of information that needs to be transferred if we want to avoid potential drawbacks—e.g., negative transfer—that could actually lead to a performance worse than the starting deep learning model remains unclear [47,48]. Thus, while a combination of transfer learning and deep learning is being widely used to attempt the solution of small data problems in the denoising of medical images [49,50,51], the reported results can still be dissatisfactory due to the lack of efficient strategies to systematically tackle these limitations [52].

The issues described above are not the only ones arising in the small data regime characteristic of CT; a statistically-significant systematic comparison and benchmarking of the supervised learning approaches can be strongly biased by so-called “concept drift”, i.e., a scenario in which the non-stationarity of the learning problem leads to a mismatch between the training data and the actual application data [53,54,55,56,57]. In CT imaging, such context-dependence of supervised ML and DL tools becomes particularly problematic when there is a discrepancy between the type of patient (age, sex, body size) and noise model tackled in the training set and those tested in the validation. This context-dependence and “concept drift” can quickly lead to unfair comparisons and unsatisfying performances of supervised learning methods. Last but not least, the robustness of the learning methods can be strongly confined by the existence of structural constraints inherent for ML and DL tools in the “small data challenge” regime; for example, while spectral filtering methods tend to outperform other unsupervised denoising algorithms [14], they also have a fundamental difficulty in dealing with high noise levels in the data [19,20]. Recently, the existence of statistically-significant overfitting boundaries has been shown empirically by employing high-performance facilities; e.g., in [58], long short-term memory (LSTM) deep neural networks [59] have been shown to systematically overfit the data and to produce results which are not statistically-significant if the condition T≥13.6D+3.8 is not satisfied (where *T* is the size of data statistics and *D* is the number of features).

While regularized time series clustering approaches have been recently demonstrated to operate in these “small data, large noise” regimes, even when the noise is an order of magnitude larger than the true signal [60,61,62,63,64,65], these studies were confined to only one-dimensional denoising problems. A systematic comparison with a broad range of supervised and unsupervised methods is still lacking. Due to the stochastic nature of the noise in CT, a statistically-significant evaluation and comparison of different CT image denoising methods has to rely on sufficiently large amounts of CT images taken from the same patient under the same combination of controls (e.g., with the same tube current and the same tube voltage). However, obtaining such an extensive set of reference-imaging data for a particular patient without a medical necessity would be unethical. A systematic comparison of methods would additionally require combining such data for multiple patients in a sampled range of patient-specific parameters (age, sex, body size, etc.) as well as for a large number of practically-relevant combinations of CT controls. Furthermore, the standard quality measures such as the Mean-Squared Error (MSE), Peak Signal-to-Noise Ratio (PSNE), and the Multi-Scale Structural Similarity Index (MS-SSIM) also rely on the availability of the reference image without noise, but generated with the same set of underlying features [66,67,68]. Finally, combining existing CT data from different sources in a metastudy is problematic as well, since the very high level of individuality of the more subtle anatomic features of the human body on a small scale [69,70] would introduce a strong bias into such a comparative study, which would also lack the reference images. Furthermore, very few datasets containing CT projection data covering the low-radiation regime are currently available in open access, mainly due to the proprietary nature of this data and the (hidden) manufacturer-specific processing of the raw data [71,72,73,74]. Even, when this information is available, as in the low-dose CT image and projection dataset described in [74], a systematic statistically-significant comparison is problematic, since for each of the patients, only a couple of images (with and without noise) are available out of the overall T=299 clinically-performed patient CT exams, and with the radiation exposures practically not going below 3 mGy. As we will show below, this ultra-low-radiation regime with radiation exposure down to 0.5 mGy and with SNR < 1.0 imposes critical challenges for the bulk of currently-available denoising methods and will receive particular attention in the tests performed below. To address these issues, we will lay two foundations in this manuscript. First, we propose a pipeline for the automated patient-specific generation of synthetic CT images, radiation exposure estimation, and LAR computation, following the strengthening movement in radiological research and using synthetic images (e.g., as in the software tool CatSim, v0.1.0) [71]. The created images are based on a data-driven estimation of CT image noise intensities and their relationship to CT control parameters [70,75,76,77]. For this purpose, we combine the LNT model for the CT-induced lifetime attributable risk [5,9,10,11,12] with the data-driven models that relate CT noise variance to the CT voltage, current, and the amount of radiation exposure [75,78]. Second, we introduce the Probabilistic formulation of the Mumford–Shah formalism (PMS) and propose a regularized Scalable Probabilistic Approximation algorithm (rSPA) and its parallel extension DD-rSPA as new methods for denoising of 3D images, comparing their computational cost and denoising performances with the state-of-the-art methods in this field. Particular focus is thereby given to investigating the possibility of reducing personalized LAR through improving denoising performance in the ultra-low-radiation regime (down to 0.1–0.5 mGy, with signal-to-noise ratios below 1.0).

## 2. Materials and Methods

### 2.1. Patient-Specific Generation of Synthetic CT Images, Radiation Exposure Estimation, and LAR Computations

In the first step of the proposed pipeline, we provide algorithms for generating synthetic noisy CT images for every relevant combination of CT control parameters, image parameters, and patient-dependent variables. Regarding the CT control parameters, we focus on the two most relevant ones that can be adjusted on the computer tomograph, which are the tube voltage, kVp, and the tube current, mA. The CT image parameters are the standard deviation of the CT quantum noise, σ, and the CT feature contrast in Hounsfield Units (HU). The patient-dependent variables for computing the overall CT-quantum noise as well as the CT-induced additional cancer risk, *r*, are the patient’s *age*, *sex*, and the subject’s size, *d*, in cm, as well as the absorbed radiation dose density ${\mathrm{CTDI}}_{\mathrm{vol}}$ in milligray (mGy).

Various approaches have been adopted in the literature to simulate the impacts of noise on the generation and analysis of CT images and applied on various levels ranging from raw data sinogram to fully-reconstructed CT images. For example, independent quantum noise was shown to affect sinogram raw CT data, from which reconstructed CT images are computed by inverting the integral Radon transform [79]. To address this issue, we model the effect of quantum noise by deploying a range of various Gaussian and non-Gaussian noises applied directly to the reconstructed images, mimicking the effect of the original quantum noise on such Radon-transformed raw data sinograms.

**Figure 1:**
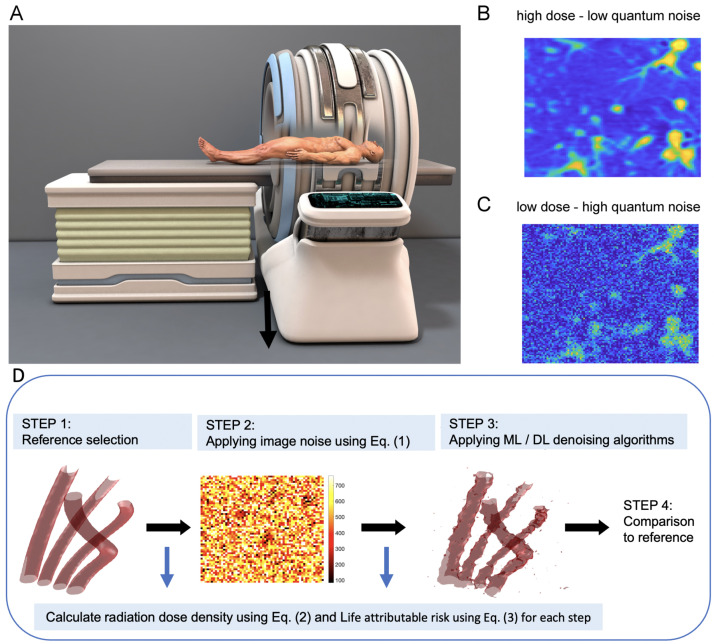
Graphical representation of our proposed pipeline workflow for automated generation and risk assessment of CT images. (**A**): Initial reference data can be either a set of real CT-data generated using high-dose radiation or artificially simulated data. (**B**) Exemplary high-quality and low-quantum noise image of lung vessels. (**C**) exemplary low-dose CT images with high quantum noise. (**D**) Workflow from image generation to subsequent benchmarking of ML/DL-denoising methods. Starting with high-quality data or artificially generated reference data, respectively, a spectrum of image noise σ is added for a multitude of combinations from patient-specific and CT control variables, as suggested in Equation (1). The noisy images are then denoised using various state-of-the-art methods and the processed images are compared to the original reference data.

The initial reference data for the automated generation of a battery of synthetic test-images can be either a set of real CT-data generated using high-dose radiation (Figure 1A) or  artificially simulated data. These reference data have to be characterized by high image quality and low quantum noise (visualized in Figure 1B), as compared to the (ultra) low-dose CT images (Figure 1C) that naturally contain a massive amount of noise and thus result in low CT-image quality. Figure 1D gives a graphical abstract of the workflow from image generation to the subsequent comparison of the various ML/DL-denoising methods based on the accuracy of the denoised image data. Starting with high-quality reference data, a broad range of typical CT image noises is imposed in a multitude of combinations from patient-specific and CT control variables. The obtained noisy CT images are subsequently denoised using various state-of-the-art methods. The denoised and segmented images are then compared to the original noiseless reference data in various performance metrics and under various CT regimes (see Section 3 for the experimental results).

To model the effect of noise in CT images, we deploy and compare three different alternatives: (i) an additive Gaussian noise model that was shown to provide an adequate description of quantum noise effects in real CT images on a small scale of several centimeters [75,80]; (ii) a non-Gaussian multiplicative noise model where the quantum noise variances change with the underlying feature color; and (iii) an empirical CT noise model sampled from the real patient data.

Computation of the noise variance σ is performed for given CT control parameters (tube current *mA*, tube voltage *kVp*), and patient-specific parameter (water-equivalent patient diameter *d*) using the non-linear regression model introduced in [75] (see Equation (1)). Equation (2) of the workflow computes the effective absorbed radiation dose density ${\mathrm{CTDI}}_{\mathrm{vol}}$ for a volume unit from the tube control parameters *mA* and *kVp* using the data-driven regression model established in [78]. Equation (3) of the image generation workflow computes the resulting lifetime attributable risk for a patient (LAR) utilizing the linear no-threshold model (LNT) proposed by the committee for Biologic Effects of Ionizing Radiation (BEIR VII) of the National Academy of Sciences of the USA [5].

(1) $$\begin{array}{c} \begin{array}{cc} ln(\sigma )= & \alpha_{0}\left(kVp\right)+\alpha_{1}\left(kVp\right)d+\alpha_{2}\left(kVp\right)ln\left(mA\right)+\alpha_{3}\left(kVp\right)d^{2} \end{array}\;\begin{array}{c} \;+\alpha_{4}\left(kVp\right)ln^{2}\left(mA\right)+\alpha_{5}\left(kVp\right)dln\left(mA\right), \end{array} \end{array}$$

(2) $$\begin{array}{cc} CTDI_{vol}= & \gamma_{0}\left(kVp,CT\;type\right)+\gamma_{1}\left(kVp,CT\;type\right)mA, \end{array}$$

(3) $$\begin{array}{cc} LAR= & \beta_{0}\left(age,sex,organ,exp.time\right)+\beta_{1}\left(age,sex,organ,exp.time\right)CTDI_{vol}. \end{array}$$

Measuring the “closeness” of synthetic images created with the noise from Equation (1) to real images—where such “closeness” is measured in a statistical sense by averaging over some sufficiently-representative statistical sample of images—would require a large number of real CT images obtained over a range of regimes, organs, patients, etc. For example, determining the optimal values for parameter functions $\alpha_{0}\left(kVp\right)$, $\alpha_{1}\left(kVp\right)$, $\alpha_{2}\left(kVp\right)$, $\alpha_{3}\left(kVp\right)$, $\alpha_{4}\left(kVp\right)$, and $\alpha_{5}\left(kVp\right)$ requires quite a substantial number of real images taken for different values of tube control variables for voltage and current [75]. Moreover, this parametrization would be specific for the particular organ and it would require new statistics of raw image data if one would want to apply it in a different CT setting. To address this problem, in the following we focus only on thorax CT imaging, where an extensive and qualitative parametrization for Equation (1) was achieved and “closeness” between real and simulated images was demonstrated [75]. A similar scenario (with a potential lack of a sufficiently-large statistics for real images) represents one of the central problems when training supervised learning methods such as neuronal networks. However, it is worth mentioning that this problem does not affect unsupervised methods such as the Probabilistic Mumford–Shah (PMS) introduced in this paper since they do not require training images for solving the denoising and segmentation problems.

In the following, we also use the data-driven parameters $\gamma_{0}$, $\gamma_{1}$, $\beta_{0}$, and $\beta_{1}$ from Equations (2) and (3), which were measured in published studies involving different CT scenarios (such as different tube voltages, currents, and exposition times), to assess the overall accumulated radiation dose and LAR [78,81].

### 2.2. Probabilistic Mumford–Shah Model Formulation

**Figure 2:**
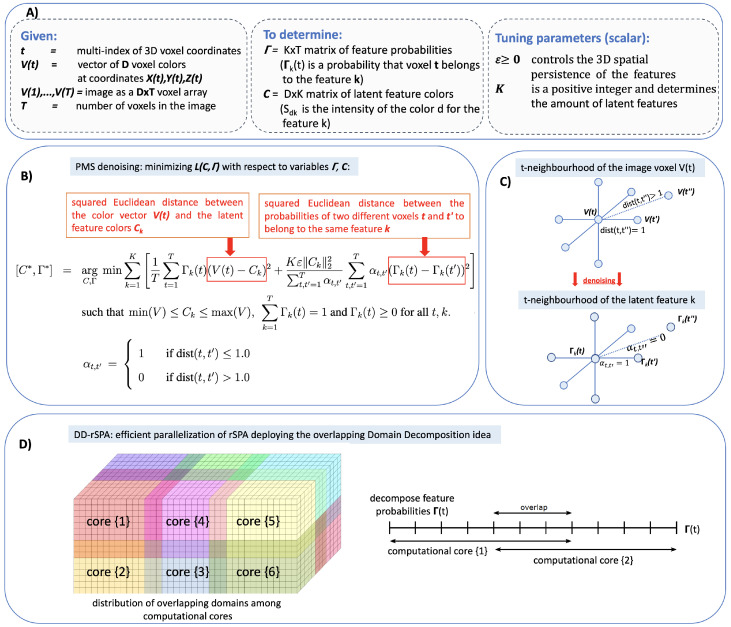
Graphical overview of the Probabilistic Mumford–Shah (PMS) framework. (**A**) Summary of the parameters and variables. (**B**) Core rSPA algorithm idea: 3D-denoising with the regularized Scalable Probabilistic Approximation algorithm (rSPA). Given the (noisy) CT voxel data *V*, rSPA minimizes the function L(C,Γ) and seeks for the optimal segmentation of *V* in terms of the *K* spatially-persistent latent features characterized by the latent feature probabilities in *K* rows of the matrix Γ, as well as by the latent colors as *K* columns of the latent color matrix *C*. Persistency of the feature segmentation is imposed by the second term of the right-hand side of the function L(C,Γ), which penalizes the differences in feature probability values in the spatially-neighboring points. (**C**) Denoising idea: latent feature probabilities are persistent (slowly-changing) 3D functions. (**D**) Graphical representation of the overlapping domain decomposition used in the parallel DD-rSPA algorithm.

In the following, we introduce the Probabilistic Mumford–Shah model formulation (PMS). More algorithmic details and a complete derivation with mathematical proofs can be found in the paper supplement. PMS (see Figure 2 for a graphical representation of the underlying algorithms) seeks a simultaneous solution of probabilistic image segmentation and noise elimination problems and aims to find the spatially most-persistent probabilistic decomposition of the image in terms of *K* latent features. Direct application of popular segmentation and clustering methods from ML to the denoising problem results in computationally-tractable tools with a favorable linear scaling of the computational cost, but also in suboptimal irregular segmentations that disregard the spatial ordering of the data [82,83,84]. Application of regularized clustering and segmentation tools that take into account the spatial ordering and regularity of the data and features (e.g., methods based on Mumford–Shah functional optimization) have unfavorable polynomial cost scaling, limiting their application to very small images or requiring very extensive computational resources [60,61,62,63,85]. In the following, we will address this key challenge with the proposed PMS rSPA algorithm (regularized Scalable Probabilistic Approximation algorithm), simultaneously achieving a qualitative (in terms of low error and sufficient spatial regularity of latent features) and computationally-tractable (linearly scalable) solution of the underlying optimization problem.

We consider a 3D image to be provided as an array $V=\left\{V(1),V(2),\cdots ,V(T)\right\}$ of *D*-dimensional patch value vectors for all *T* of three-dimensional CT voxels, with patch values $V\left(t\right)\in R^{D}$ being, for example, the grey-color intensities $V_{d}\left(t\right),d=1,\cdots ,D$ of the *D*-dimensional voxel patch with an index *t*. Without a loss of generality, in the following applications, we will consider the common grayscale CT images with one-voxel patches (D=1) and *T* being of the order $10^{5}–10^{7}$. The problem of denoising can then be considered as a numerical problem of searching for *K D*-dimensional latent features characterized by *K D*-dimensional distinct feature vectors $\left\{C_{1,k},\cdots ,C_{D,k}\right\}$, with *k* taking values between 1 and *K*. Spatial characteristics of these *K* latent features we will be searching for will be provided by (a priori unknown) latent feature probabilities $\Gamma_{k}\left(t\right)$, representing the probabilities of an actual (noisy) voxel V(t) to belong to a particular latent (noiseless) feature with an index *k*. Such a numerical procedure can be performed by a broad range of clustering and segmentation algorithms from ML (e.g., K-means, scalable probabilistic approximation, and others) [82,83,84,86]. For example, one possibility would be to minimize the sum of the errors $L_{t}\left(C,\Gamma \left(t\right)\right)$ when approximating every vector V(t) with its probabilistic representation ${\tilde{V}}_{C,\Gamma }\left(t\right)=\sum_{k=1}^{K}\Gamma_{k}\left(t\right)C_{k}$:

(4) $$\begin{array}{ccc} \left[C^{*},\Gamma^{*}\right] & = & \underset{C,\Gamma }{arg min}\frac{1}{T}\sum_{t=1}^{T}L_{t}\left(C,\Gamma \left(t\right)\right), \end{array}$$

where $L_{t}\left(C,\Gamma \left(t\right)\right)=∥V\left(t\right)-{\tilde{V}}_{C,\Gamma }\left(t\right){)∥}_{2}^{2}$. It is straightforward to see that when *C* is fixed, the solution of minimization problem (4) is equivalent to *T* independent minimizations of individual errors $L_{t}$ with respect to their particular Γ(t), and can be performed independently for each *t*. This allows a very efficient, independent, and parallel numerical treatment of Equation (4) and results in a favorable linear scaling of the computational cost with growing size and dimension of the data [86]. The downside of this independent and additive structure of optimization problem (4) is that it results in solutions that are independent of any spatial permutation of the original data *V*, since the right-hand side of Equation (4) is clearly invariant with respect to any arbitrary re-ordering of the summation indices *t*. This indicates that the solutions of such an optimization problem will not change if we arbitrarily change the spatial ordering of the voxels in the original image. This invariance of the clustering outcomes with respect to the data ordering is a common characteristic of a broad class of ML methods, including, for example, K means and fuzzy-K means clustering-methods that belong to the most popular ML algorithms, with over 3 million citations according to Google-Scholar [86]. While analyzing spatially-ordered data, in addition to a simple segmentation (4) of the image into *K* latent probabilistic features, we would like to enforce a spatial persistence of the underlying features. To achieve this, we can force any two voxel points V(t) and $V(t^{\prime })$ to have similar latent probabilities of belonging to the same features if their positions are close enough to each other. In order to deal with the relative position of the voxels, we can use the kernel function, a very popular concept in ML. The simplest alternative to measure the “closeness” of two different voxels would be provided by the Euclidean kernel, defined as a distance function $\alpha_{t,t^{\prime }}$ between two distinct points with indices *t* and $t^{\prime }$:

(5) $$\begin{array}{c} \alpha_{t,t^{\prime }}=\left\{\begin{array}{cc} 1 & \;\mathrm{if}\;{\mathrm{dist}}^{\mathrm{Eucl}}\left(t,t^{\prime }\right)\leq \alpha_{0},\\ 0 & \;\mathrm{if}\;{\mathrm{dist}}^{\mathrm{Eucl}}\left(t,t^{\prime }\right)>\alpha_{0}, \end{array}\right. \end{array}$$

where $\alpha_{0}$ is some user-defined threshold (e.g., $\alpha_{0}=1$ in this paper’s applications).

Then, following idea behind the Mumford–Shah functional formulation [85], the spatially-persistent optimal probabilistic approximation ${\tilde{V}}_{C^{*},\Gamma^{*}}$ of the original image data *V* can be computed via the numerical minimization of the regularized form of the original clustering problem Equation (4):

(6) $$\begin{array}{ccc} \left[C^{*},\Gamma^{*}\right] & = & \underset{C,\Gamma }{arg min}\frac{1}{T}\left[\sum_{t=1}^{T}L_{t}+\frac{\bar{\varepsilon }}{\sum_{t,t^{\prime }=1}^{T}\alpha_{t,t^{\prime }}}\sum_{t,t^{\prime }=1}^{T}\alpha_{t,t^{\prime }}∥{\tilde{V}}_{C,\Gamma }\left(t\right)-{\tilde{V}}_{C,\Gamma }\left(t^{\prime }\right){)∥}_{2}^{2}\right]. \end{array}$$

The second term in the right-hand side of this functional controls the spatial regularity and smoothness of the obtained solutions. Please note that, in contrast to the original clustering problem Equation (4), Equation (6) is not invariant with respect to permutations of *V*, and allows the obtaining spatially-regular solutions $\left[C^{*},\Gamma^{*}\right]$, with the persistence growing when increasing the scalar control parameter $\bar{\varepsilon }$. However, these features of the regularized problem come at the price of losing the very favorable linear scalability of the computational cost of Equation (4); optimization with respect to different Γ(t) cannot be performed independently when *C* is fixed, unlike what happens in the case of clustering problems such as SPA (4), where one solves T independent *K*-dimensional optimization problems for Γ(t) with fixed *C*. The second term in Equation (6)—aimed at enforcing spatial regularity and persistence—at the same time introduces global coupling between different Γ(t) and requires the solution of very large coupled KT-dimensional nonlinear optimization problems [60,61,85]. This confines the applicability of the image analysis methods based on Equation (6), when working on common hardware (e.g., workstations), to relatively-small images, with KT not larger then 50,000–100,000 [60,61]. The direct solution of Equation (6), as well as indirect Bayesian solutions of Equation (6) based on Markov Chain Monte Carlo sampling (MCMC), are costly beyond 1D and would require extensive use of High-Performance Computing facilities (HPC) for large realistic 3D images with $KT\approx 10^{5}–10^{7}$ [63,87].

One of the key methodological insights of this work is that one can systematically derive an exact upper bound approximation of the regularized Equation (6) that can be solved with a linearly scalable and parallelizable numerical algorithm for realistic 3D images (with $10^{6}–10^{7}$ voxels), while requiring few minutes on a common laptop:

(7) $$\begin{array}{c} \begin{array}{cc} \; & \left[C^{*},\Gamma^{*}\right]=\underset{C,\Gamma }{\arg}\;\min\sum_{k=1}^{K}\left[\frac{1}{T}\sum_{t=1}^{T}\Gamma_{k}\left(t\right){∥V\left(t\right)-C_{k}∥}^{2}\right.\\ & \;\left.+\frac{K\varepsilon ∥C_{k}{∥}^{2}}{\sum_{t,t^{\prime }=1}^{T}\alpha_{t,t^{\prime }}}\sum_{t,t^{\prime }=1}^{T}\alpha_{t,t^{\prime }}{\left(\Gamma_{k}\left(t\right)-\Gamma_{k}\left(t^{\prime }\right)\right)}^{2}\right],\\ \; & \mathrm{such}\;\mathrm{that}\;\min\left(V\right)\leq C_{k}\leq \max\left(V\right),\;\sum_{k=1}^{T}\Gamma_{k}\left(t\right)=1\;\mathrm{and}\;\Gamma_{k}\left(t\right)\geq 0\;\mathrm{for}\;\mathrm{all}\;t,k. \end{array} \end{array}$$

As proven in Lemma S1 of the paper supplement, solutions of Equation (7) are also exact solutions of the original regularized problem Equation (6) if the segmentations are discrete (i.e., if $\Gamma_{k}\left(t\right)$ take only discrete values 0 or 1). These solutions provide upper bound approximate minimizers of Equation (6) if $\Gamma_{k}\left(t\right)$ take fuzzy values between 0 and 1. In contrast to the original clustering SPA-functional Equation (4), Equation (7) has Γ(t) outside of the norm in the first (clustering) term and the analytical structure of the second (regularizing) term is very different from the structure that one would obtain by directly deploying common regularization tools (such as Ridge, Lasso, and elastic net regularizations) to the original clustering problem (4). Applying Ridge, Lasso and elastic net regularizations with respect to both variables *C* and Γ in problem (4) would result in regularization terms of the form $+\epsilon_{C}∥C_{k}∥+\epsilon_{\Gamma }∥\Gamma_{k}∥$ and would require tuning at least the two regularization parameters $\epsilon_{C}$ and $\epsilon_{\Gamma }$.

The numerical solution of the obtained optimization problem Equation (7) can be computed with the monotonically-convergent rSPA algorithm: starting with some arbitrarily chosen *K* feature vectors *C*, one iterates between solving the above problem for Γ (with fixed *C*) and minimizing (7) for *C* (with fixed Γ). As proven in Lemma S2, S3 and in Theorem S1 of the paper supplement, rSPA always results in the monotonic minimization of (7), with a linear iteration cost scaling O(KDT). The rSPA algorithm can be efficiently parallelized by deploying the Domain Decomposition idea (DD) widely used in various areas. A graphical representation of the idea underlying the resulting parallel DD-rSPA algorithm is shown in Figure 2, while a detailed description of the DD-rSPA algorithm is provided in Section 2 of the paper supplement. Commented computer code implementing both algorithms is provided for open access at https://www.dropbox.com/sh/rw4t6ydkpi64w8y/AAA9katysG09w7ljsvUqPwwna?dl=0 (accessed on 18 March 2022) and can be run on a laptop with a MATLAB installation. Numerical tests on noisy images with different sizes and noise levels reveal that the overall computational cost of both the sequential rSPA and parallel DD-rSPA algorithms grows linearly with image size and with decreasing signal-to-noise ratios (corresponding to increasing noise levels), as we can see in Section 3.

### 2.3. Relation of Probabilistic Mumford–Shah and rSPA Algorithm to Regularized Mumford–Shah Framework (MS) and Rudin–Osher–Fatemi (ROF) Total Variation Model

The Mumford–Shah formalism originally introduced in [85] is one of the most well-understood and elaborated theoretical and algorithmic frameworks for edge-preserving image denoising. It aims to find an optimally-denoised image $V^{d}$ that is simultaneously smooth and close enough to the original noisy image *V*. Then, keeping the previously introduced notation, in the most common discrete Mumford–Shah formulation such a denoised image $V^{d}$ can be found as a solution to the following optimization problem:

(8) $$\begin{array}{ccc} \left[V^{d}\right] & = & \underset{V^{d}}{\arg}\;\min\frac{1}{T}\sum_{t=1}^{T}\left[∥V\left(t\right)-V^{d}{\left(t\right)∥}^{2}+\epsilon^{2}∥\nabla V^{d}\left(t\right)∥\right], \end{array}$$

where the first term measures the “closeness” of the original and the denoised images, and the second term regularizes the “smoothness” of the denoised image by penalizing the norm of its average gradient. One of the key theoretical insights to this problem (8) was provided in the work by Rudin, Osher, and Fatemi [88]; by deploying the Euler–Lagrange principle, they showed that the solution to the minimization problem (8) is equivalent to solving a parabolic Partial Differential Equation (PDE). This opened a way for applying very efficient PDE solvers and the so-called level-set methods to the image denoising problem. The numerical solution of both the original MS-formulation (8) and of the PDE-based ROF-formalism is commonly achieved by deploying the Galerkin ansatz:

(9) $$\begin{array}{ccc} V^{d}\left(t\right) & = & \sum_{k=1}^{K}C_{k}\Gamma_{k}\left(t\right), \end{array}$$

where $\Gamma_{k}\left(t\right)$ is a fixed set of known basis functions (e.g., mesh functions, finite element functions, wavelet basis functions, Fourier basis functions, etc.) and $C_{k}$ are the unknown coefficients that are found numerically [88,89,90,91,92,93].

The most important difference between the Probabilistic Mumford–Shah (PMS) problem formulation (7) and the common MS and ROF methods is the form of the Galerkin expansion (9); the PMS problem (7) deploys the probabilistic expansion (9), with *C* and Γ(t) being a priori unknown *non-parametric* probability density vectors, whereas common MS and ROF tools dwell on a priori fixed *parametric* sets of non-probabilistic basis functions Γ. Hence, in contrast to the parametric optimization problem (8) that allows a straightforward Euler–Lagrange reformulation in form of the parabolic PDE, the introduced PMS-formulation deals with a non-parametric variational problem (7) subject to both equality and inequality constraints that does not allow a straightforward Euler–Lagrange reformulation and does not allow the deployment of the aforementioned very efficient algorithms from PDE numerics for its solution. One of the central methodological developments of this manuscript consists in showing that, despite this presumed limitation, it is possible to efficiently solve the PMS problem numerically (7) with an iterative algorithm that has a linear scalability of the computational cost. A direct numerical comparison of PMS to the common MS- and ROF-tools [88,93] reveals very significant differences in denoising performance, cost, and parallel scalability (see the results in Section 3).

### 2.4. Practical Implementation

#### 2.4.1. Synthetic CT Image Generation Model

To create the additive Gaussian CT noise, we used the parameter value *‘gaussian’*, while the non-Gaussian multiplicative noise images were created by using the function imnoise() with the parameter value *‘speckle’*. The parameter σ is, in both cases, selected according to the description below. The MATLAB code implementing this CT image generation workflow is available at https://www.dropbox.com/sh/rr0no9vdo8osx44/AAAHQxXJnxT8P0LPs7wTRBv7a?dl=0 (accessed on 18 March 2022). Generation of the nonparametric empirical CT noise was implemented in the function create_CT_image_noise() available at https://www.dropbox.com/s/xbwwrk9y2napgpy/create_CT_image_noise.m?dl=0 (accessed on 18 March 2022).

#### 2.4.2. Common CT Image Denoising and Image Quality Assessment Methods

We used the same software platform (MATLAB, version: 2021b (The MathWorks, Inc., Natick, MA, USA)) and the same hardware (Mac workstation with 28 CPU cores (Apple Inc., Cupertino, CA, USA)) for all calculations to guarantee a fair comparison of the denoising methods and to rule-out software- and platform-induced differences that could bias this comparison. All deployed common denoising and image quality assessment tools are available in the MATLAB functions from the “Image Processing”, “Deep Learning”, “Machine Learning”, and “Wavelets” toolboxes of MathWorks. We used denoising methods based on local window filtering of the data (3D Gaussian filtering with the MATLAB function imgaussfilt3(), 3D local median filtering with the MATLAB function medfilt3() and bilateral filtering with the MATLAB function imbilatfilt()) [14,16,17,18], spectral denoising methods (the 3D wavelets denoising with the MATLAB function wavedec3()) [15,19,20,21,22] and a deep learning denoising method based on pre-trained feed-forward denoising convolutional neural networks (DnCNNs, with the MATLAB functions denoiseImage() and denoisingNetwork()) [13,25,26,27].

## 3. Results and Discussion

### 3.1. Application and Comparison of the PMS Model with Standard Methods

**Figure 3:**
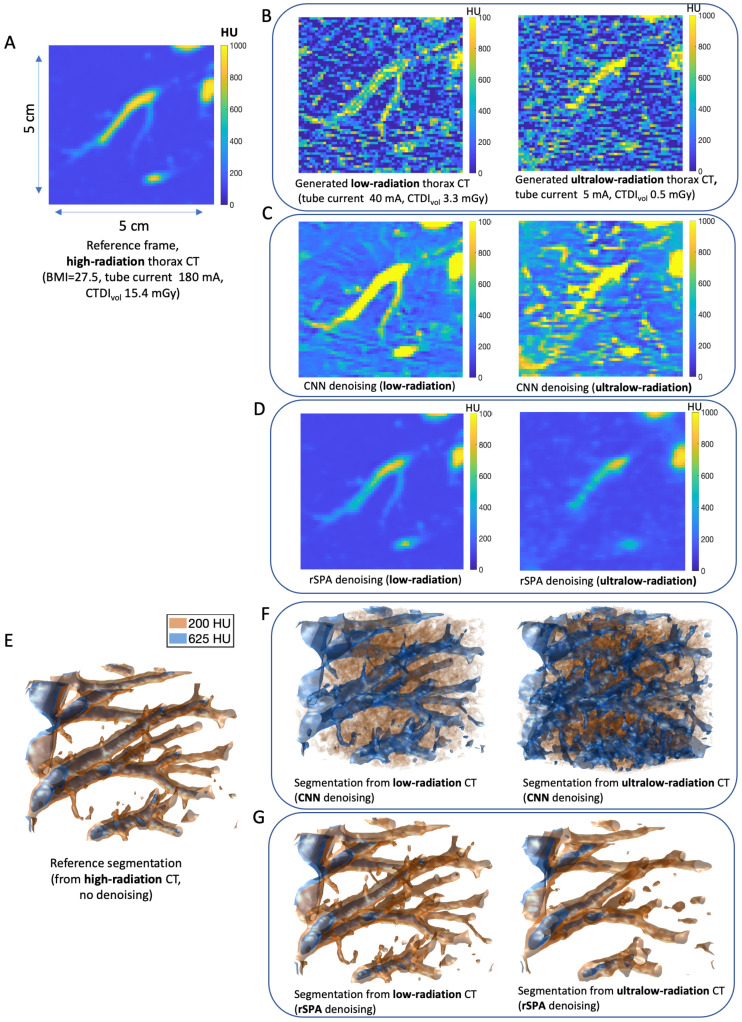
Radiation exposure, quantum noise, and denoising performance of CNNs and rSPA in low-radiation and ultra-low-radiation thorax CT regimes. (**A**) Reference data of a thorax CT voxel fragment (approx. 5 cm${}^{3}$) of a 19-year-old female with the BMI 27.5, acquired with the Somatum Emotion 16 2007 (Siemens Aktiengesellschaft, Berlin, Germany) at 130 kV tube voltage. (**B**) Simulated decrease in the radiation exposure ${\mathrm{CTDI}}_{\mathrm{vol}}$ from 15.6 mGy (reference frame) to 3.3 mGy (for low-radiation simulations) and 0.5 mGy (ultra-low-radiation) results in a significant increase of quantum noise. (**C**) Reconstructed images using CNNs. (**D**) Reconstructed images using rSPA. (**E**) 3D segmentation of the original reference frame. (**F**) 3D segmentation based on the images denoised using CNNs. (**G**) 3D-segmentation of the images denoised by rSPA.

Next, we compare the denoising performance for a broad selection of supervised and unsupervised algorithms using the synthetic CT images generated with the pipeline introduced above. As a noiseless CT reference, we first use the patient data exemplified in Figure 3A. It has 274,625 voxels and represents a cubic CT area of around 5 cm × 5 cm × 5 cm. The  data came from a high-radiation CT (180 mA tube current, ${\mathrm{CTDI}}_{\mathrm{vol}}$ 15.4 mGy, section from a thorax CT of a 19-year-old female patient).

For each particular combination of tube-specific and patient-specific parameters, we used this reference image to create statistics of 100 different independent noisy synthetic CT images for every parameter combination. Figure 3B shows the increase in noise when reducing the radiation exposure. In the following, we use the state-of-the art version of the ML, AI, and image processing toolboxes of MathWorks from 2021 to compare the performance of all of the commonly-available denoising and segmentation algorithms in these toolboxes to the PMS algorithm introduced in this paper. To illustrate the performance of DL on these data, we first apply one of the most widely-used DL denoising networks: the Convolutional Neuronal Network DnCNN-3 from [25], with over 3264 citations according to Google Scholar. It was trained on a comprehensive collection of imaging datasets (including the Berkeley segmentation dataset, with over a million image pairs for training) with a very broad range of signal-to-noise ratios and noise types (both Gaussian and non-Gaussian). Figure 3C,D show the effects of denoising by DL DnCNN-3 from [25] and rSPA, respectively. in low- and ultra-low-radiation CT. Figure 3E shows a 3-dimensional segmentation obtained from a stack of such high-radiation CT data, whereas Figure 3F,G give the segmentation based on the images denoised using DnCNN-3 and rSPA, respectively. Figure 3E,F are all obtained from two feature isosurfaces at 625 and 200 Hounsfield Units (HU), respectively, representing the interior of blood vessels in the lung volume segment.

Apparently, rSPA provides denoised images and segmentations that are much closer to the high-radiation reference images. In particular, we observe that as the noise increases, DL denoising methods start recognizing features from noise artifacts that were not part of the true reference images. As already mentioned above, such deterioration of the performance of ML and DL methods can be attributed to various reasons, including, on the one hand, the insufficient training data set and the “small data challenge” [39,40,41,42] and, on the other hand, the effect of “concept drift” stemming from the mismatch between the type of image features and noise model used during the model training and the noise model in the validation data [53,54,55].

**Figure 4:**
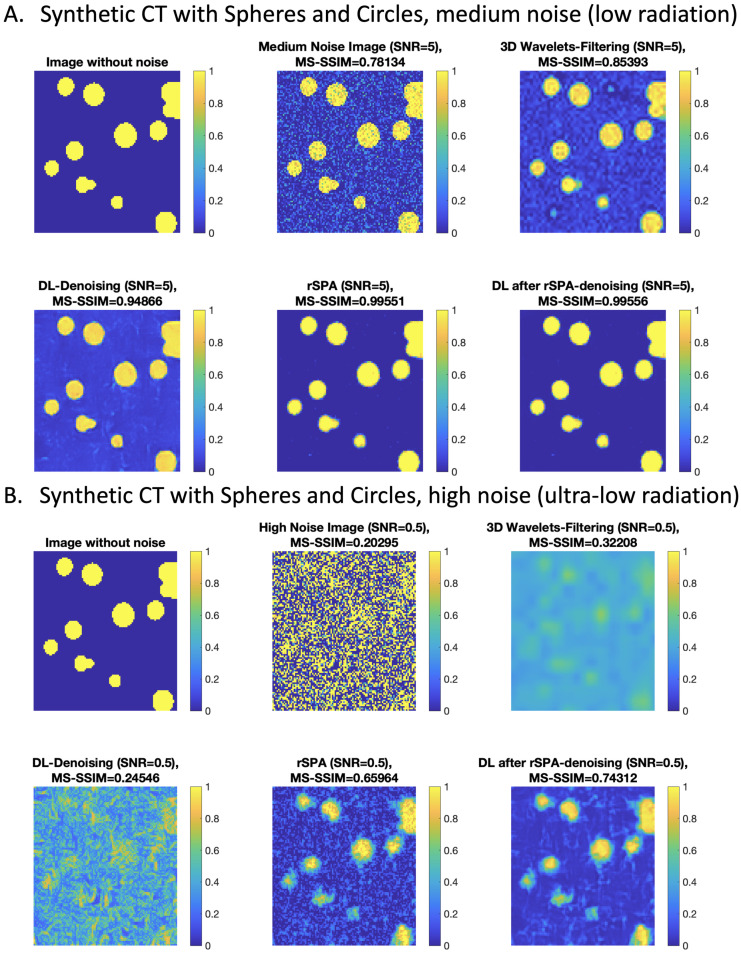
Comparing denoising performance on synthetic CT images of noisy circles, with DL from Figure 3 additionally trained to recognize circles for non-Gaussian noise model: (**A**) medium noise scenario, corresponding to low-radiation regime with around 3.3 mGy; (**B**) high noise scenario, corresponding to ultra-low-radiation regime with 0.5 mGy.

To discern the potential impact of “concept drift” and to rule-out the possibility that the “hallucinations” observed for DL CNN in Figure 3 in the ultra-low-radiation regime are induced by the insufficient size of the training dataset, we additionally train the DnCNN-3 from [25] first with 10,000 image pairs (with and without noise) of spheres and circles of various sizes, and then with further 40,000 image pairs. We performed this two-stage training procedure to evaluate the performance improvement induced by providing more training data. The complete additional training took around 8 days on a machine with 28 CPUs (Intel Xeon Gold 6240R 2.4G, 14C/28T (Intel Corporation, Santa Clara, CA, USA)) and 384 GB RAM (DDR4-2933) using up to 90% of the physical cores and ∼120 GB of memory. The resulting denoising network is provided for open access at https://www.dropbox.com/s/ia69h9fhgud2vpt/additionallytrained_DnCNN-3_network.mat?dl=0 (accessed on 18 March 2022). We found that using a larger training dataset (with further 40,000 image pairs) can only bring negligible improvements, confirming the earlier finding reported in [25]. The noisy images in every pair were created using the empirically sampled non-Gaussian CT noise at various levels, covering low- and ultra-low-radiation regimes (down to 0.2 mGy, corresponding to signal-to-noise ratios between 5 and 0.1). In Figure 4, we show some of the results obtained from the application of the additionally trained DnCNN to the noisy images of circles and spheres that were not used in the training by deploying the same empirically-sampled non-Gaussian CT noise model used in the training at the medium noise level (SNR = 5, corresponding to the low-radiation CT) and at the high noise level (SNR = 0.5, corresponding to the ultra-low-radiation CT). Complete comparisons are provided as movie files and are available at https://www.dropbox.com/sh/n2dbl4h9p4o0p92/AABRkAalhXoaiKFO7ixsSzKga?dl=0 (accessed on 18 March 2022). In Figure 4, we observe the same effect of a quick deterioration of DL denoising quality with the increasing noise as in Figure 3; at the medium noise level, DL provides high-quality denoising, outperforming a very popular unsupervised 3D wavelets denoising tool [15,19,20,21,22]. However, at high noise levels, DL is outperformed by the 3D wavelet denoising. Interestingly, the best performance, in both cases, is achieved when applying the DL denoising to the data that has been previously denoised by rSPA.

Making an interim assessment of these results, we can conclude that the deteriorating performance of DL denoising is neither a result of “concept drift” (since the type of features and the noise model deployed in the training and in validation were the same), nor a consequence of the training data set insufficiency (since we observed only negligible performance improvements of DL when expanding the additional training data from 10 K to 50 K image pairs). A possible explanation can be given by the fact that here we observe a fundamental robustness boundary of DL denoising in the high noise regime, similar to the Donoho-boundary for wavelets methods [19,20]. As we will see in the following, further numerical results provided below give additional support to this hypothesis.

**Figure 5:**
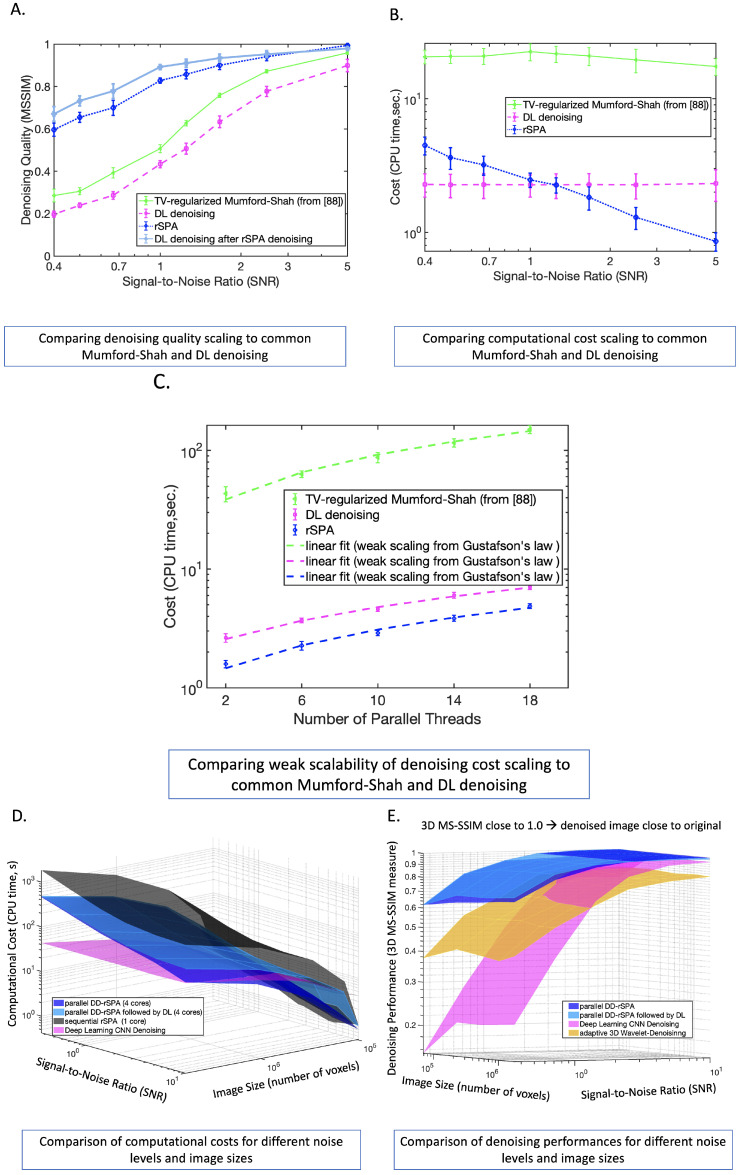
Comparing denoising quality, cost and parallelizability: (**A**–**C**) comparison of PMS rSPA algorithm to the regularized Mumford–Shah denoising tool introduced in [93] and to the additionally trained DL denoising algorithm from Figure 3 and Figure 4; (**D**,**E**) computational cost scaling and performance for DL (without taking into account time for additional training), sequential rSPA, parallel DD-rSPA and DD-rSPA followed by DL. Each point of each method’s curve and surface is obtained from statistical averaging of the respective values obtained by analyzing 10 randomly-generated images with these particular combinations of image size and noise level.

In the next step, we compare the computational cost scaling, denoising performance scaling, and parallelizability scalings for DL, TV-regularized Mumford–Shah denoising from [93], sequential rSPA, parallel DD-rSPA, and parallel DD-rSPA followed by DL. We are particularly interested in analyzing the dependence of these characteristics on the image size and noise intensity. For every combination of image size and noise level, we create 10 randomly-generated images of spheres and circles with the non-Gaussian noise, matching the characteristics of the additionally trained DnCNN-3 to avoid bias through “concept drift”. The code reproducing these results is available at https://www.dropbox.com/sh/6p3q62zaelcyugz/AACkEjggyKcIAdgtoHGWClWPa?dl=0 (accessed on 18 March 2022). The results are summarized in Figure 5; their computation required around 30 h on a laptop with a MacBook Pro 3.1 GHz Quad-Core Intel Core i7 (4 cores) with 16 GB RAM. The measurement of the computational cost for DL considered only the pure time of applying the fully-trained DL network to a noisy image and did not include the time needed for the additional training (that was around 8 days on the workstation, as mentioned above). As can be seen in Figure 5, for all considered methods, the overall cost scales linearly with the image size, while parallel DD-rSPA demonstrates the weak scaling of parallel computation cost (see Figure 5C). DD-rSPA allows the denoising of a 3D image with $10^{7}–10^{8}$ voxels in the ultra-low-radiation regime (SNR=0.5) at around 3–10 min on a MacBook Pro laptop with four cores. Interestingly, the costs of DL and common MS-denoising practically do not depend on the noise level, whereas the cost of rSPA and DD-rSPA grows linearly with the decreasing SNR. According to Theorem S1 of the paper supplement, the iteration cost of rSPA and DD-rSPA does not depend on the noise intensity; this linear dependence of the overall cost on noise is solely explained by the linear increase in the number of rSPA and DD-rSPA iterations required to achieve the solution of the minimization problem (7) with the linearly reducing SNR. In other words, these results show that DL and common MS-denoising invest the same amount of work at different noise levels, whereas rSPA and DD-rSPA invest work linearly-proportional to the SNR and increase with the relative increase in the noise. A comparison of the denoising quality scalings in Figure 5 provides additional evidence towards the hypothesis formulated above; the deterioration of the denoising performance of DL in the area of large noise (small SNR) and smaller image sizes—where DL is outperformed by the 3D wavelet denoising—is neither the result of an insufficient training dataset nor of “concept drift”, but can be explained through the existence of a fundamental robustness boundary for DL denoising in the high noise regimes (with SNR < 1.0). Indeed, the scaling of DL performance decay observed in Figure 5 exhibits a much steeper robustness boundary than the Donoho-boundary [19,20] of the wavelets denoising robustness (compare magenta and orange surfaces in Figure 5E). This finding is also confirmed by inspecting the performance of DL when it is applied to the images that were previously denoised by DD-rSPA (light blue surface in Figure 5B). This combination of unsupervised DD-rSPA followed by supervised DL exhibits the best performance among all the considered methods in this high noise regime.

**Figure 6:**
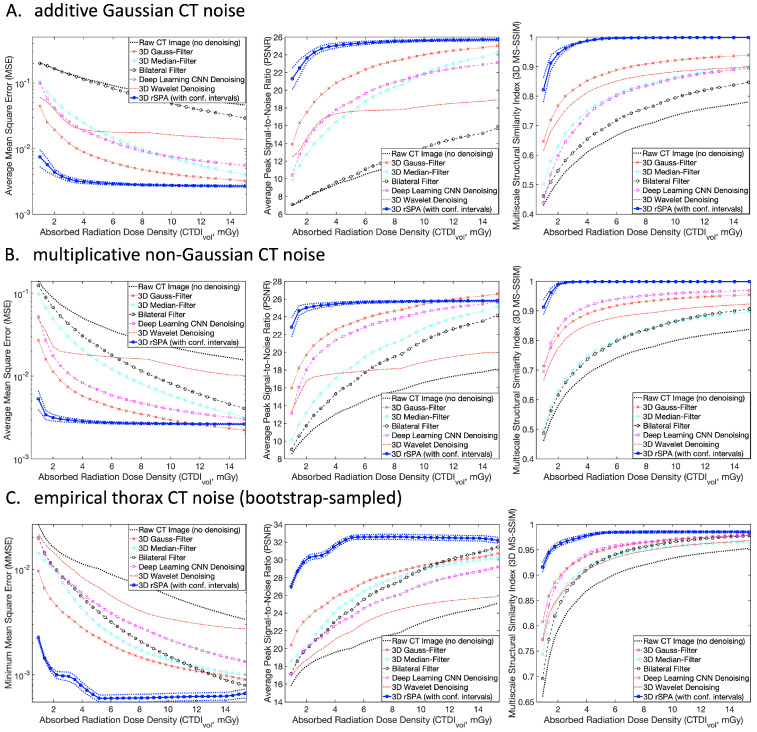
Comparing CT image denoising performances for three CT noise models: (**A**) additive Gaussian noise model (CT noise variance is independent of the feature color); (**B**) multiplicative non-Gaussian noise model (CT noise variance changes with the amplitude of the underlying color signal); (**C**) empirical noise obtained from the thorax CT patient data. In (**A**,**B**), generation of synthetic images was performed for a patient with a water-equivalent diameter of 30 cm, which is subject to a Thorax CT with a typical tube voltage of 120 kV in the range of tube currents between 5–180 mA and a set of artificial anatomic features from Figure 2A (with a feature contrast of 200 HU). In (**C**), real patient data were used. Comparison is performed with three primary image quality criteria: with mean squared error (left panels); with peak signal-to-noise ratio (middle panels); and with the 3D multiscale structural similarity index (right panels).

Next, from synthetic CT images generated from circles and spheres, we return to the analysis of CT images generated from real anatomical features. Using the CT image generation and LAR-assessment pipeline, we compare the performance of denoising methods with a broad range of absorbed radiation dose densities. This comparison is made for two synthetic noise models (Figure 6A,B, with Gaussian and non-Gaussian noise) and for the empirical nonparametric CT noise model obtained from the real patient data (Figure 6C). The results of this comparison are shown in terms of three major image quality measures. As expected, the Gaussian 3D filtering exhibits the best performance among the common tools for all three additive Gaussian noise scenarios from Figure 6A. On the other hand, the non-Gaussian deep learning DnCNN denoising outperforms the other tools (except rSPA) in the non-Gaussian and empirical noise situations, as can be seen in Figure 6B,C. However, in the overall comparison, the rSPA method markedly outperforms all considered denoising tools in all image quality measures for all three noise models. As can be seen from Figure 6, rSPA achieves the same quality of the denoised image obtained with DnCNN (3D MS-SSIM around 0.9) with around 15-fold smaller absorbed radiation dose density (${\mathrm{CTDI}}_{\mathrm{vol}}=0.95\;\mathrm{mGy}$ for rSPA vs. ${\mathrm{CTDI}}_{\mathrm{vol}}=15\;\mathrm{mGy}$ for DnCNN).

**Figure 7:**
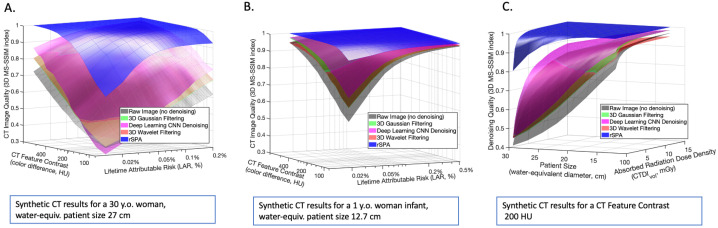
Comparing denoising methods with the average Multiscale Structural Similarity Index (3D MS-SSIM): (**A**) varying the true underlying feature contrast and LAR for a synthetic 30-year-old female patient with a water-equiv. cross-section of 27 cm; (**B**) varying the true underlying feature contrast and LAR for a synthetic 1-year-old female infant patient with a water-equiv. cross-section of 12.7 cm; (**C**) denoising performance comparison when varying the patient size and the effective absorbed radiation dose density, with the 200 Hounsfield Units (HU) feature contrast differences.

In Figure 7, we compare the average denoising performances measured with the 3D MS-SSIM image quality measure for a range of practically-relevant CT feature color intensity differences, lifetime attributable risks (LAR), and absorbed radiation dose densities. These results again demonstrate that rSPA is superior to all other considered tools in all analyzed regimes. The 3DMS-SSIM of the blue surfaces corresponding to rSPA is close to 1.0 almost everywhere, thus indicating that the denoised images are very close to the reference CT images without noise. The powerful effect of image quality-preserving LAR reduction by denoising, especially in female infants, is visible in Figure 7B. Furthermore, we can notice the substantial LAR reduction obtained through the application of the rSPA algorithm. in particular in case of the infant patient. Indeed, Denoising with rSPA achieves the same image quality obtained with DnCNN (3D MS-SSIM around 0.97 for feature color differences around 50–100 HU), but with a 22.6-fold smaller LAR (LAR=0.015% for rSPA vs. LAR=0.34% for DnCNN). In Table 1, we report, for the different methods, a comparison of the image quality loss caused by an increasing reduction of the lifetime attributable risk in the case of the infant patient and with a fixed value of the feature contrast at 200 HU.

**Figure 8:**
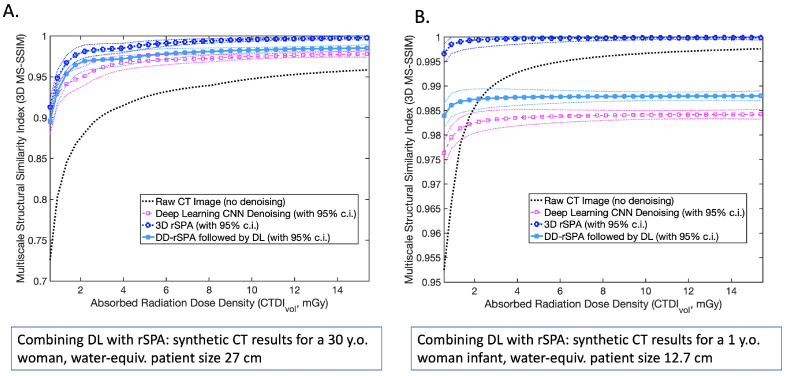
Comparing denoising methods with the average Multiscale Structural Similarity Index (3D MS-SSIM) for simulated thorax CT: (**A**) varying the absorbed radiation dose for a synthetic 30-year-old female patient with a water-equiv. cross-section of 27 cm; (**B**) varying the absorbed radiation dose for a synthetic 1-year-old female infant patient with a water-equiv. cross-section of 12.7 cm. Noiseless thorax CT image used as reference in this performance comparison is available at https://www.dropbox.com/s/29x0xivg8l80q10/female_lung_thorax_CT_image_section_v2.mat?dl=0 (accessed on 18 March 2022). Dotted lines show 95% nonparametric confidence intervals (c.i.) obtained for every value of ${\mathrm{CTDI}}_{\mathrm{vol}}$ from 100 different independently-generated noisy synthetic CT images, using the MATLAB function *quantile()*.

Finally, in Figure 8, we evaluate the performance of DL with and without preliminary DD-rSPA denoising, comparing it to the denoising performance of DD-rSPA for the synthetic noisy CT images generated with real anatomic features from thorax CT. The noiseless thorax CT image used as reference in this performance comparison is available at https://www.dropbox.com/s/29x0xivg8l80q10/female_lung_thorax_CT_image_section_v2.mat?dl=0 (accessed on 18 March 2022). The dotted lines show 95% nonparametric confidence intervals (c.i.) obtained for every value of ${\mathrm{CTDI}}_{\mathrm{vol}}$ from 100 different independently-generated noisy synthetic CT images, using the MATLAB function *quantile()*. These results support our previous findings; applying DL to the image previously denoised with DD-rSPA provides a statistically-significant improvement of DL denoising performance.

### 3.2. Implementation Details

For each of the considered images, the standard deviation of the local Gaussian smoothing kernel σ was changed in the range $\sigma =\left[0.2,0.4,0.6,\cdots ,2\right]$. The value leading to the least MSE deviation between the denoised and the original CT image was taken to compute the curves in Figure 4 and Figure 5. Similarly, for the optimal 3D wavelet filtering, all of the wavelet bases available in MATLAB were checked for all of the possible depths of level decompositions and the wavelet decomposition with the minimal MSE error was selected. Pre-training of DnCNN was done with over 20 million images and was provided in the “Deep Learning Toolbox”. The image quality measures plotted in Figure 4 and Figure 5 were computed using the MATLAB functions from the “Image Processing Toolbox”: 3D mean-squared error (MSE) [66] was computed as the average over the 2D MSE errors obtained with the MATLAB function immse(); 3D Peak Signal-to-Noise Ratio (PSNR) was obtained as an average over the 2D PSNR image error measures [67] implemented in the MATLAB function psnr(); 3D Multi-Scale Structural Similarity Index Measure (3D MS-SSIM) [68] was obtained with the 3D image volume measure MATLAB function multissim3().

The curves in Figure 6, Figure 7 and Figure 8 show averages over individual denoising results obtained for 100 different independently-generated noisy synthetic CT images that were obtained for every particular combination of tube-specific and patient specific parameters. In Figure 5, the surfaces represent averages over 10 randomly-realized noisy CT images. To provide a fair comparison, the same random CT image realizations were used with every denoising method. The dotted lines in Figure 6 and Figure 8 show the 95% nonparametric confidence intervals (c.i.) computed with the MATLAB function quantile().

## 4. Conclusions

We introduced an algorithmic pipeline for the generation of synthetic patient-specific CT images and radiation-induced risk assessment. We used it to compare various CT image denoising approaches in a range of practically-relevant CT regimes. The ultra-low-radiation CT regime represents a three-fold challenge for all the standard denoising methods since: (i) the reduction in the radiation exposure leads to a substantial increase in the noise level, eventually making it impossible for the standard unsupervised and spectral denoising tools to distinguish the noise from the underlying true image signal; (ii) the heterogeneity of individual anatomical features, patient sizes, and CT conditions, together with the lack of training data, can lead to the “concept drift” problem, thus making the identification of some pre-trained features and patterns in the noisy CT images particularly difficult; (iii) in this context, even the performance of one of the most popular supervised denoising CNNs, trained in a wide range of noise regimes [25], quickly deteriorates.

To tackle these challenges, we introduced the Probabilistic Mumford–Shah formalism (PMS) (7) and showed that it can be efficiently solved numerically through the unsupervised regularized Scalable Probabilistic Approximation method (rSPA), which seeks a simultaneous solution to the image segmentation and noise elimination problems. We proved that it provides a computationally-cheap (with a linear cost scaling, see Figure 5, Lemma S1–S3 and Theorem S1 of the paper supplement) exact upper bound approximation of the numerically much more expensive regularized probabilistic segmentation problem (6). We also introduced DD-rSPA, a parallel extension of the rSPA algorithm based on the decomposition of the 3D domain in overlapping subdomains. Commented code for both algorithms was provided for open access. Numerical tests on images with different sizes and noise levels revealed that: (i) the overall computational cost grows linearly with the image size and with a decrease in the signal-to-noise ratio (SNR) for both the sequential rSPA and the parallel DD-rSPA algorithms, while the common Mumford–Shah, 3D wavelets, and DL denoising tools require the same computational effort regardless of the image SNR; (ii) the observed deterioration in the performance of DL denoising is neither the result of “concept drift”, nor a consequence of the limited size of the training set. Further tests on artificial and real data (Gaussian and non-Gaussian, with additive, multiplicative, and nonparametric empirical CT noise, with continuous and discontinuous feature boundaries) showed that rSPA outperforms all the other considered denoising methods in a wide array of performance measures. The linear scaling of the parallel DD-rSPA algorithm allows using a normal laptop for tasks that would otherwise require extensive hardware (e.g., workstations and HPC facilities). Indeed, with DD-rSPA it is possible to obtain a high-quality denoising (with **3DMS-SSIM** around 0.9) of a 3D image with $10^{7}$ voxels in the ultra-low-radiation regime (SNR = 0.5) in only 3 min on a MacBook Pro laptop with 4 cores. None of the other denoising methods considered were able to come close to this performance.

Using rSPA and DD-rSPA creates the opportunity to obtain a significant patient-specific reduction of the radiation-imposed risks, allowing a 20-fold estimated LAR reduction for infants and a 10-fold LAR reduction for adults. According to the risk assessment protocol introduced in [10], these results from Figure 7B suggest that applying this personalized denoising methodology to ultra-low-radiation pediatric CTs might lead to the prevention of around 90% of the deadly cancers they induce (i.e., ∼11,000 cases every year worldwide).

We showed that the DD-rSPA algorithm can be used to generate a statistically-significant increase in the performance of the other DL and ML methods that have been recently developed. Indeed, many of the existing tools were trained in regimes with moderate and low noise levels, and a preliminary unsupervised denoising step with DD-rSPA can extend their applicability to the ultra-low-radiation regime, where the noise level is significantly higher. Furthermore, the particular design of rSPA and DD-rSPA aims to simultaneously tackle the denoising and the segmentation of noisy 3D images by solving an unsupervised learning problem, while allowing the optimal and linearly-scalable “smooth” segmentation of the denoised image in 3D. This approach is different from the one adopted by the majority of the available tools, which are focused exclusively on the denoising part and lack the segmentation component.

Among possible other application areas of the sequential rSPA and the parallel DD-rSPA algorithms, we can find the denoising and segmentation of ultra-noisy 2D and 3D movie data. In the case of 2D movies, the time component can be considered as a third image dimension in the rSPA algorithm. Another possible application area—i.e., 3D movies—emerges, for example, in fMRI applications pertaining to various biomedical areas (e.g., in cardiology), where the main challenge consists in detecting the moving boundary of the inner organ and in distinguishing it from other eventual shapes in a time-resolved noisy dynamics [94] (Some examples of movie denoising with DD-rSPA are available at https://www.dropbox.com/sh/n2dbl4h9p4o0p92/AABRkAalhXoaiKFO7ixsSzKga?dl=0 (accessed on 18 March 2022)). Finally, beyond CT data denoising and segmentation, we also see direct application possibilities for other imaging techniques such as fiber-optic fluorescence imaging, diffusion tensor imaging, and large-scale 3D segmentation tasks with electron microscopy images.

## Figures and Tables

**Table 1:** Deterioration of CT image quality (decrease in 3D MS-SSIM index, baseline = 100%) caused by a reduction of lifetime attributable risk (LAR) for different methods. The CT scans pertain to the infant patient, with a fixed feature contrast of 200 HU.

	Image Quality Loss (3D MS-SSIM, in %)				
**Reduction of** **LAR (in %)**	**Raw Image**	**3D Gaussian** **Filtering**	**DL CNN** **Denoising**	**3D Wavelet** **Filtering**	**rSPA**
6	1.16	0.77	0.54	0.79	0.01
16	1.26	0.84	0.59	0.86	0.01
23	1.34	0.90	0.63	0.92	0.01
29	1.44	0.96	0.67	0.99	0.01
36	1.56	1.04	0.73	1.07	0.01
42	1.70	1.13	0.79	1.16	0.01
49	1.88	1.25	0.88	1.29	0.01
56	2.12	1.41	0.99	1.45	0.02
63	2.45	1.63	1.14	1.67	0.02
70	2.92	1.95	1.36	2.00	0.02
77	3.69	2.46	1.72	2.52	0.03
83	5.11	3.40	2.38	3.49	0.04
90	8.72	5.81	4.07	5.97	0.06
97	25.96	18.20	13.37	18.16	0.17

## Data Availability

Code is available for open access at https://www.dropbox.com/sh/rw4t6ydkpi64w8y/AAA9katysG09w7ljsvUqPwwna?dl=0 (accessed on 18 March 2022) under the BSD 3-Clause License.

## Supplementary Materials

The following are available online at https://www.mdpi.com/article/10.3390/jimaging8060156/s1. References [63,85,95,96,97,98,99] are cited in the supplementary materials.
