# Supplementary material for: Low-Cost Probabilistic 3D Denoising with Applications for Ultra-Low-Radiation Computed Tomography
Source: J Imaging. 2022 May 31;8(6):156. doi: 10.3390/jimaging8060156 (PMC9224620; doi:10.3390/jimaging8060156)
Supplement: Supplementary file 1 [file jimaging-08-00156-s001.zip › jimaging-1668547-supplementary.pdf]

Supplementary material for the manuscript entitled: Low-cost probabilistic 3D denoising with applications to Ultra-Low Radiation Computed Tomography

Illia Horenko, Lukas Pospisil, Edoardo Vecchi, Steffen Albrecht, Alexander Gerber, Beate Rehbock, Albrecht Stroh, and Susanne Gerber

This document provides supplementary material for the manuscript entitled: Order of magnitude risk reduction in Computed Tomography with the unsupervised machine learning denoising. In particular, we provide the complete mathematical formulation of 3D regularized Scalable Probabilistic Approximation (rSPA) optimization problem and present:

- **Lemma S1** - derivation of the rSPA problem formulation,
- **Algorithm S1** - a subspace algorithm for solving rSPA optimization problem in pseudo-code. The algorithm consists of two consequent inner optimization problems; namely so-called C-problem and Γ-problem.
- **Lemma S2** - the solvability and the computational cost of solving the C-problem,
- **Lemma S3** - the solvability and the computational cost of solving the Γ-problem,
- **Theorem S1** - the properties and the computational cost of solving rSPA problem. The proof is based on Lemma 2 and Lemma 3.

1 Regularized Scalable Probabilistic Approximation Algorithm (rSPA)

Formulation: Let $t \in \mathbb{N}^3$ be a multi-index of 3D voxel coordinates and

$$V := [V(1), \dots, V(T)] \in \mathbb{R}^{D,T}$$

be a 3D CT image represented as a matrix of given D-dimensional voxel colours at 3D coordinates $X := [X(1), \dots, X(T)] \in \mathbb{R}^{3,T}$.

We will be searching for a probabilistic approximation $\tilde{V}_{C,\Gamma}$ of the image in terms of K latent features characterized by K distinct color vectors $\{C_{1,k}, \dots, C_{D,k}\}$, with k taking values between 1 and K. Spatial characteristics of these K latent features that we will be searching for will be provided by (a priori unknown) latent feature probabilities $\Gamma_k(t)$, being the probabilities of an actual (noisy) voxel $V(t)$ to belong to a particular latent (noiseless) feature with an index k:

$$\tilde{V}_{C,\Gamma} := [\sum_{k=1}^{K} \Gamma_k(1) C_k, \dots, \sum_{k=1}^{K} \Gamma_k(T) C_k] \in \mathbb{R}^{D,T}$$

Then, following the idea behind the Mumford-Shah functional formulation [6], spatially-persistent optimal probabilistic approximation $\tilde{V}_{C^*,\Gamma^*}$ of the original image data V can be computed via the numerical minimization of the function:

$$\begin{aligned}
[C^*, \Gamma^*] &= \arg_{C,\Gamma} \min_{\Gamma \in \Omega_\Gamma, C \in \Omega_C} \mathcal{L}(C, \Gamma) = \\
&= \arg_{C,\Gamma} \min_{\Gamma \in \Omega_\Gamma, C \in \Omega_C} \left[\frac{1}{T} \sum_{t=1}^{T} \mathrm{dist}^2(V(t), \tilde{V}_{C,\Gamma}(t)) + \frac{\bar{\varepsilon}}{\sum_{t,t'=1}^{T} \alpha_{t,t'}} \sum_{t,t'=1}^{T} \alpha_{t,t'} \mathrm{dist}^2(\tilde{V}_{C,\Gamma}(t), \tilde{V}_{C,\Gamma}(t')) \right],
\end{aligned} \tag{1}$$

where $\mathrm{dist}^2(\cdot, \cdot)$ is a square of some distance (e.g., Euclidean distance, l_1-distance, etc.), feasible sets are given as

$$\Omega_\Gamma = \{\Gamma = [\Gamma(1), \dots, \Gamma(T)] \in \mathbb{R}^{K,T} : \ \Gamma \geq 0 \text{ and } \forall t : \sum_{k=1}^{K} \Gamma_k(t) = 1\}, \tag{2}$$

$$\Omega_C = \{C = [C_1, \dots, C_K] \in \mathbb{R}^{D,K} : \ \min(V) \leq C \leq \max(V)\}, \tag{3}$$

and function $\alpha_{t,t'}$ is the indicator function of the voxel neighborhood defined as

$$\alpha_{t,t'} = \begin{cases} 1 & \text{if } \mathrm{dist}(X(t), X(t')) \leq \alpha_0, \\ 0 & \text{if } \mathrm{dist}(X(t), X(t')) > \alpha_0, \end{cases}$$

with $\bar{\varepsilon} \geq 0$ and $\alpha_0 > 0$ being some user-defined parameters.

Lemma S1. *(rSPA as an approximate upper bound formulation for probabilistic segmentations with Eu-clidean distance) Approximate solutions of the problem (1,2,3) with Eulidean distance*

$$\mathrm{dist}(x, y) = \|x - y\|_2, \tag{4}$$

can be found minimizing its upper bound

$$\begin{aligned}
[C^*, \Gamma^*] &= \arg_{C,\Gamma} \min_{\Gamma \in \Omega_\Gamma, C \in \Omega_C} L(C, \Gamma) = \\
&= \arg_{C,\Gamma} \min_{\Gamma \in \Omega_\Gamma, C \in \Omega_C} \sum_{k=1}^{K} \left[\frac{1}{T} \sum_{t=1}^{T} \Gamma_k(t) \|V(t) - C_k\|_2^2 + \frac{K \bar{\varepsilon} \|C_k\|_2^2}{\sum_{t,t'=1}^{T} \alpha_{t,t'}} \sum_{t,t'=1}^{T} \alpha_{t,t'} (\Gamma_k(t) - \Gamma_k(t'))^2 \right].
\end{aligned} \tag{5}$$

Moreover, $L(C, \Gamma) \geq \mathcal{L}(C, \Gamma)$ *(for all* C, Γ *).*

Proof. Since the square of any norm is a convex function and for any $t = 1, \dots, T$ coefficients $\Gamma_k(t)$ forms the coefficients of convex combination, we can apply Jensens inequality to the first term of (1) and obtain

$$\mathrm{dist}^2(V(t), \tilde{V}_{C,\Gamma}(t)) = \left\| V(t) - \sum_{k=1}^{K} \Gamma_k(t) C_k \right\|_2^2 = \left\| \sum_{k=1}^{K} \Gamma_k(t) \left(V(t) - C_k \right) \right\|_2^2 \leq \sum_{k=1}^{K} \Gamma_k(t) \left\| V(t) - C_k \right\|_2^2 .$$

In the case of the second term, we use the properties of the sum of squares

$$\begin{aligned} \forall u \in \mathbb{R}^K : \quad & \left(\sum_{k=1}^{K} u_k \right)^2 \leq K \sum_{k=1}^{K} u_k^2, \\ \forall v \in \mathbb{R}^D, \forall \alpha \in \mathbb{R} : \quad & \left(\sum_{d=1}^{D} \alpha v_d \right)^2 = \alpha^2 \left(\sum_{d=1}^{D} v_d \right)^2, \end{aligned}$$

and we get

$$\begin{aligned} \mathrm{dist}^2(\tilde{V}_{C,\Gamma}(t), \tilde{V}_{C,\Gamma}(t')) &= \left\| \left(\sum_{k=1}^{K} \Gamma_k(t) C_k \right) - \left(\sum_{k=1}^{K} \Gamma_k(t') C_k \right) \right\|_2^2 = \left\| \sum_{k=1}^{K} \left(\Gamma_k(t) - \Gamma_k(t') \right) C_k \right\|_2^2 \\ &\leq K \sum_{k=1}^{K} \left\| \left(\Gamma_k(t) - \Gamma_k(t') \right) C_k \right\|_2^2 = K \sum_{k=1}^{K} \left(\Gamma_k(t) - \Gamma_k(t') \right)^2 \left\| C_k \right\|_2^2 . \end{aligned}$$

□

Algorithm: Approximate solutions of the optimization problem (5,2,3) can be found using the iterative subspace algorithm, i.e., it is solved as a sequence of split optimization problems, see Algorithm S1.

Let V be given voxel data, K be a fixed number of latent features, and $\bar{\varepsilon} \geq 0$ be a priori chosen regularization parameter.
Choose a feasible initial approximation $\Gamma^0 \in \Omega_\Gamma$ and set iteration counter $\mathrm{it} = 0$.

while $\| L(C, \Gamma^{\mathrm{it}}) - L(C^{\mathrm{it}-1}, \Gamma^{\mathrm{it}-1}) \|$ *is not sufficiently small*

*solve the problem with fixed $\Gamma^{\mathrm{it}-1}$ (C-**problem**)*

$$C^{\mathrm{it}} = \arg_{C} \min_{C \in \Omega_C} L(C, \Gamma^{\mathrm{it}-1}) \tag{6}$$

*solve the problem with fixed C^{it} (Γ-**problem**)*

$$\Gamma^{\mathrm{it}} = \arg_{\Gamma} \min_{\Gamma \in \Omega_\Gamma} L(C^{\mathrm{it}}, \Gamma) \tag{7}$$

$\mathrm{it} = \mathrm{it} + 1$
endwhile

Return an approximation of the latent features color C^{it} and an approximation of latent feature affiliation probability vectors Γ^{it}.

Algorithm S1: Regularized Scalable Probabilistic Approximation algorithm (rSPA).

Lemma S2. *(The properties of C-problem (6))*

1. *the optimization problem (6) has always solution,*
2. *(6) is a box-constrained convex Quadratic Programming problem (QP) with diagonal Hessian matrix and it has analytical solution,*
3. *evaluation of analytical solution for solving problem (6) is $\mathcal{O}(TKD)$.*

Proof.

1. Let $\Gamma = \hat{\Gamma}$ be fixed. We are dealing with minimization problem with continuous convex objective function on closed set, therefore by Weierstrass Extreme value theorem [3], the problem has always solution.

2. The objective function of problem (5) with Euclidean measure (4) is given by

$$L(C, \hat{\Gamma}) = \sum_{k=1}^{K} \left[\left(\frac{1}{T} \sum_{t=1}^{T} \hat{\Gamma}_k(t) \| V(t) - C_k \|_2^2 \right) + \kappa_k \| C_k \|^2 \right] \tag{8}$$

where we denoted constant

$$\kappa_k = \frac{\bar{\varepsilon}}{\sum_{t,t'=1}^{T} \alpha_{t,t'}} \sum_{t,t'=1}^{T} \alpha_{t,t'} (\hat{\Gamma}_k(t) - \hat{\Gamma}_k(t'))^2, \quad k = 1, \ldots, K. \tag{9}$$

Since the minimization of (8) with respect to (3) is separable in $k = 1, \ldots, K$, we have

$$C_k^* = \arg_{C_k} \min_{C_k \in \Omega_{C_k}} \left(\frac{1}{T} \sum_{t=1}^{T} \hat{\Gamma}_k(t) \| V(t) - C_k \|_2^2 \right) + \kappa_k \| C_k \|^2 = \arg_{C_k} \min_{C_k \in \Omega_{C_k}} \underbrace{\frac{1}{2} C_k^T A_k C_k - C_k^T b_k}_{= f_k(C_k)} \tag{10}$$

with

$$\Omega_{C_k} = \{ C_k \in \mathbb{R}^D : \quad \min(V) \leq C_k \leq \max(V) \}$$

and

$$A_k = \sigma_k I_D, \quad \sigma_k = \left(\frac{1}{T} \sum_{t=1}^{T} \hat{\Gamma}_k(t) \right) + \kappa_k, \quad b_k = \sum_{t=1}^{T} \hat{\Gamma}_k(t) V(t), \tag{11}$$

where $I_D \in \mathbb{R}^D$ is identity matrix. Please, notice that for any non-empty cluster (i.e., $\sum\limits_{t=1}^{T} \Gamma_k(t) > 0$) $\sigma_k > 0$ and therefore (10) is stricly convex optimization problem on closed convex set and consequently (10) has unique solution. If the cluster is empty, then (10) can be simplified to

$$C_k^* = \arg_{C_k} \min_{C_k \in \Omega_{C_k}} 0,$$

which has infinite number of solutions, i.e., any $C_k^* \in \Omega_{C_k}$ solves the problem.

The problem (10) is (again) separable in $d = 1, \ldots, D$ and we can write

$$\begin{aligned} C_{d,k}^* &= \arg_{C_{d,k}} \min_{C_{d,k} \in \Omega_{C_{d,k}}} \tfrac{1}{2} \sigma_k C_{d,k}^2 - b_{d,k} C_{d_k} = \arg_{C_{d,k}} \min_{C_{d,k} \in \Omega_{C_{d,k}}} \tfrac{1}{2} C_{d,k}^2 - \tfrac{b_{d,k}}{\sigma_k} C_{d_k} \\ &= \arg_{C_{d,k}} \min_{C_{d,k} \in \Omega_{C_{d,k}}} \| C_{d,k} - \tfrac{b_{d,k}}{\sigma_k} \|_2 = P_{\Omega_{C_{d,k}}} \left(\tfrac{b_{d,k}}{\sigma_k} \right) \end{aligned}$$

with interval

$$\Omega_{C_{d,k}} = [\min(V), \max(V)] \subset \mathbb{R}$$

and projection onto this interval $P_{\Omega_{C_{d,k}}} : \mathbb{R} \to \Omega_{C_{d,k}}$ given by

$$P_{\Omega_{C_{d,k}}}(\tau) := \min \{ \max(V), \max \{ \min(V), \tau \} \}. \tag{12}$$

3. The computation of K sums of T vectors of dimension D in (11) and (9) followed by the computation of projection (12) is $\mathcal{O}(TKD)$.

□

Lemma S3. *(The properties of* Γ*-problem* (7)*)*

1. *Problem* (7) *is a convex QP on separable simplexes (i.e., with bound inequality and linear equality constraints).*

2. *Assembling the objects of problem* (7) *is* $\mathcal{O}(TKD)$.

3. *One iteration of Spectral Projected Gradient method for QP (SPG-QP, [7]) for solving problem* (7) *is* $\mathcal{O}(TK)$.

Proof.

1. Let $C = \hat{C}$ be fixed. The objective function of problem (5) with Euclidean measure (4) is given by

$$L(\hat{C}, \Gamma) = \sum_{k=1}^{K} \left[-\left(\sum_{t=1}^{T} \hat{\Gamma}_k(t) w_{k,t} \right) + \xi_k \sum_{t,t'=1}^{T} \alpha_{t,t'} \left(\Gamma_k(t) - \Gamma_k(t') \right)^2 \right] \tag{13}$$

with constants

$$w_{k,t} = -\frac{1}{T} \| V(t) - \hat{C}_k \|_2^2, \quad \xi_k = \frac{\bar{\varepsilon} \operatorname{dist}^2(C_k, 0)}{\sum_{t,t'=1}^{T} \alpha_{t,t'}}. \tag{14}$$

In the following, we simplify the objective function (13) into the standard QP form.

Let us denote the diagonalization of matrix into vector

$$\gamma = \operatorname{vec}(\Gamma^T),$$

and introduce multi-index (t,k) of vector γ by

$$\gamma_{(t,k)} = \gamma_{(k-1)T+t} = \Gamma_k(t),$$

where γ_j is j-th component of $\gamma \in \mathbb{R}^{KT}$. At first, notice that quadratic term

$$\alpha_{t,t'}(\Gamma_k(t) - \Gamma_k(t'))^2 = \alpha_{t,t'}[\Gamma_k(t), \Gamma_k(t')] \begin{bmatrix} 1 & -1 \\ -1 & 1 \end{bmatrix} \begin{bmatrix} \Gamma_k(t) \\ \Gamma_k(t') \end{bmatrix} = \gamma_{(:,k)}^T H(t,t') \gamma_{(:,k)},$$

where $\gamma_{(:,k)} = [\gamma_{(1,k)}, \dots, \gamma_{(T,k)}]^T \in \mathbb{R}^T$ and the components of matrix $H(t,t') \in \mathbb{R}^{T,T}$ are given by

$$H_{t_1,t_2}(t,t') = \begin{cases} \alpha_{t,t'} & \text{if } t_1 = t \text{ and } t_2 = t, \\ \alpha_{t,t'} & \text{if } t_1 = t' \text{ and } t_2 = t', \\ -\alpha_{t,t'} & \text{if } t_1 = t \text{ and } t_2 = t', \\ -\alpha_{t,t'} & \text{if } t_1 = t' \text{ and } t_2 = t, \\ 0 & \text{elsewhere.} \end{cases}$$

Using this notation, we are able to simplify the quadratic term of (13) into

$$\sum_{k=1}^{K} \xi_k \sum_{t=1}^{T} \sum_{t'=1}^{T} \alpha_{t,t'} (\Gamma_k(t) - \Gamma_k(t'))^2 = \sum_{k=1}^{K} \xi_k \gamma_{(:,k)}^T \underbrace{\left(\sum_{t=1}^{T} \sum_{t'=1}^{T} H(t,t') \right)}_{=\hat{A}} \gamma_{(:,k)} = \frac{1}{2} \gamma^T \underbrace{\left(\Xi \otimes \left(2\hat{A} \right) \right)}_{=A} \gamma,$$

where $\Xi = \operatorname{diag}(\xi_1, \dots, \xi_K) \in \mathbb{R}^{K,K}$ is diagonal matrix and $\otimes$ denotes matrix Kronecker product. Matrix $A \in \mathbb{R}^{KT,KT}$ is a block-diagonal matrix of K diagonal blocks $2\xi_k \hat{A} \in \mathbb{R}^{T,T}$. Let us remark that the matrix

$$\sum_{t'=1}^{T} H(t,t')$$

forms the Laplace matrix corresponding to graph of neighborhood of vortex t (stencil). Consequently, matrix $\hat{A}$ is composed from contributions from all stencils constructed in vortexes in the system. Such a matrix is symmetric positive semidefinite.

The objective function can be written in the form of convex quadratic function

$$L(\hat{S}, \Gamma) = \frac{1}{2} \gamma^T A \gamma - w^T \gamma. \tag{15}$$

The feasible set (2) defines the lower bound constraints and equality constraints of the optimization problem. This feasible set is closed and convex, the objective function (15) is continuous, therefore using the Weierstrass Extreme value theorem [3], the optimization problem has always solution.

2. Before solving the QP problem (15), we assemble the Hessian matrix A, linear term b, and constraints Ω_Γ (2). The assembly of the linear term (14), where one has to sum TK values of distance functions between vectors of dimension D. If the complexity of chosen distance function evaluation is $\mathcal{O}(D)$, then the overall complexity is $\mathcal{O}(TKD)$.

3. Spectral Projected Gradient method for QP (SPG-QP, [7]) is an iterative algorithm for solving minimization problems of convex quadratic function $f(x) := \frac{1}{2}x^T Ax - b^T x, f : \mathbb{R}^n \rightarrow \mathbb{R}$ on closed convex feasible set $\Omega \subset \mathbb{R}^n$ defined by separable constraints with simple projections

$$P_\Omega(x) = \arg\min_{y\in\Omega} \|x - y\|. \quad (16)$$

From the initial approximation $x^0 \in \Omega$, the process is generating the approximations x^{it} by

$$x^{\text{it}+1} = x^{\text{it}} + \beta_{\text{it}} d^{\text{it}}, \quad (17)$$

where $d^{\text{it}} \in \mathbb{R}^n$ is *projected gradient* computed as

$$d^{\text{it}} = x^{\text{it}} - P_\Omega(x - \alpha_{\text{it}} \nabla f(x^{\text{it}})). \quad (18)$$

The step-size α_{it} is computed by Barzilai-Borwein rule [1]

$$\alpha_{\text{it}} = \frac{\langle x^{\text{it}} - x^{\text{it}-1}, x^{\text{it}} - x^{\text{it}-1}\rangle}{\langle \nabla f(x^{\text{it}}) - \nabla f(x^{\text{it}-1}), x^{\text{it}} - x^{\text{it}-1}\rangle} \quad (19)$$

and the step-size β_{it} is a result of Grippo, Lampariello, and Lucidi line-search method [5] for satisfying so-called *generalized Armijo criteria*

$$f(x^{\text{it}} + \beta_{\text{it}} d^{\text{it}}) < f_{\max} \tau \beta_{\text{it}} \langle \nabla f(x^{\text{it}}), d^{\text{it}}\rangle \quad (20)$$

with safeguarding parameter $\tau \in (0, 1)$ and $f_{\max}$ is a maximum function value in previous $m \geq 1$ iterations. The original SPG algorithm has been proposed by [2] for solving general optimization problems and the convergence is based on satisfaction of condition (20). Recently, [7] show that in the case of quadratic objective function, the line-search algorithm can be replaced by direct formula which satysfies (20)

$$\beta^{\text{it}} = \min\left\{1, (1-\tau)\xi + \sqrt{(1-\tau)^2\xi^2 + \frac{2(f_{\max} - f(x^{\text{it}}))}{\langle Ad^{\text{it}}, d^{\text{it}}\rangle}}\right\} \quad \text{with} \quad \xi = -\frac{\langle \nabla f(x^{\text{it}}), d^{\text{it}}\rangle}{\langle Ad^{\text{it}}, d^{\text{it}}\rangle}. \quad (21)$$

The algorithm non-monotonically decreases the norm of projected gradient and the function value until the stopping criteria is satisfied.

In the general case, the most time-consuming operation is the multiplication by Hessian matrix A, all other computations includes the evaluation of scalar products. In our case, the matrix has a special pattern; it is a block-diagonal matrix of K diagonal blocks of band matrices. Computational complexity of multiplication with such a matrix is $\mathcal{O}(KT)$. Please notice that the feasible set (2) is separable in T and the projection onto the set can be computed independently for each column of matrix Γ

$$\Gamma_{:,t} \in \{\gamma \in \mathbb{R}^K : \quad \gamma \geq 0 \text{ and } \sum_{k=1}^{K} \gamma_k = 1\}.$$

The projection onto each individual *simplex* is $\mathcal{O}(K)$, [4].

Summing up all the operations performed during one iteration of SPG-QP algorithm, the overall computation complexity is $\mathcal{O}(TK)$.

□

Theorem S1. *(The computational complexity of Algorithm 1)*

1. *Algorithm 1 generates the approximations with monotonically non-increasing objective function.*
2. *Let* dist *be Eulidean distance* (4)*. One iteration of Algorithm 1 is* $\mathcal{O}(TKD)$*.*

Proof.

1. Since the iterations solves the optimization problems (6) and (7), we have

$$\forall C \in \mathbb{R}^{D,K} : L(C,\Gamma^{\text{it}-1}) \geq L(C^{\text{it}},\Gamma^{\text{it}-1}) \quad \text{and} \quad \forall \Gamma \in \Omega_\Gamma : L(C^{\text{it}},\Gamma) \geq L(C^{\text{it}},\Gamma^{\text{it}}).$$

   Choosing $C = C^{\text{it}-1}$ and $\Gamma = \Gamma^{\text{it}-1}$, we get

$$L(C^{\text{it}-1},\Gamma^{\text{it}-1}) \geq L(C^{\text{it}},\Gamma^{\text{it}-1}) \geq L(C^{\text{it}},\Gamma^{\text{it}}).$$

2. The statement is the consequence of Lemma 2 and Lemma 3.

□

2 Parallel Regularized Scalable Probabilistic Approximation Algorithm based on overlapping Domain Decomposition (DD-rSPA)

In the case of the computation in real-world applications, we are dealing with two main challenges: the computational demand (the number of operations that have to be performed to obtain the solution) and the memory limitation (the amount of information which can be processed by given machine). Both of these issues can be solved by High-Performance Computing (HPC). In this case, the algorithm runs on the machine which consists of several computational units (cores, processors, graphics processing unit) which are operating with distributed memory. The computational capacity of the largest supercomputers in the world can achieve more than 10^{17} FLOPS (floating-point operations per second) and can operate with several petabytes of memory. However, the massively parallel architectures cannot be efficiently utilized without appropriate massively parallel algorithms. For example in the case of discretized solution of partial differential equations with a huge number of variables, the original problem can be decomposed into smaller independent subproblems using so-called Domain Decomposition methods (DD). The idea is to solve subproblems in parallel using the individual computational units of the machine (i.e., nodes, cores, GPUs) and the only limitations arise in the case of global communication for the satisfaction of the continuity of global solution through domains. In practice, two different approaches are commonly used: overlapping DD, where the subdomains overlap by more than the interface (e.g., Schwarz alternating method or additive Schwarz method), and non-overlapping methods, where the subdomains intersect only on their interface (e.g., Balancing domain decomposition (BDDC), or Finite Element Tearing and Interconnecting (FETI)).

To analyze the problem of the global continuity and non-separability, suppose that we decompose the solution into two disjoint parts $\Gamma_{\{1\}}$ and $\Gamma_{\{2\}}$. Then the objective function of corresponding quadratic optimization Γ-problem (15) can be written as (after appropriate permutation of indexes)

$$\begin{aligned} f(\gamma) = f(\gamma_{\{1\}},\gamma_{\{2\}}) &= \tfrac{1}{2}[\gamma_{\{1\}}^T,\gamma_{\{2\}}^T] \begin{bmatrix} A_{\{1,1\}} & A_{\{1,2\}} \\ A_{\{1,2\}}^T & A_{\{2,2\}} \end{bmatrix} \begin{bmatrix} \gamma_{\{1\}} \\ \gamma_{\{2\}} \end{bmatrix} - [w_{\{1\}}^T, w_{\{2\}}^T] \begin{bmatrix} \gamma_{\{1\}} \\ \gamma_{\{2\}} \end{bmatrix} \\ &= \underbrace{\frac{1}{2}\gamma_{\{1\}}^T A_{\{1,1\}}\gamma_{\{1\}} - w_{\{1\}}^T\gamma_{\{1\}}}_{=f_{\{1\}}(\gamma_{\{1\}})} + \underbrace{\frac{1}{2}\gamma_{\{2\}}^T A_{\{2,2\}}\gamma_{\{2\}} - w_{\{2\}}^T\gamma_{\{2\}}}_{=f_{\{2\}}(\gamma_{\{2\}})} + \gamma_{\{1\}}^T A_{\{1,2\}}\gamma_{\{2\}}. \end{aligned} \tag{22}$$

Using this equality, we can observe that the original minimization problem is separable into two disjoint minimization problems except the coupling term $\gamma_{\{1\}}^T A_{\{1,2\}}\gamma_{\{2\}}$. If we solve the problem separably to obtain $\gamma_{\{1\}}$ and $\gamma_{\{2\}}$ on separated computational units, we have to additionally handle with this term.

In our case, we implement the Schwartz Domain Decomposition method and separate the domain into overlapping domains, see Figure S1. For the demonstration, the figure presents the DD into two domains in 1D, but the approach is easily extendable to 3D and multiple domains in each direction, see Figure S2.

In the first step of algorithm, we solve the problem in each domain separately, i.e., we solve corresponding QP problem with appropriate block of the Hessian matrix and the block of linear term. Each domain sends the solution in overlap to the neighbouring domains and this vector is used for the computation of coupling term in local objective function. This operation can be written in terms of (22) -

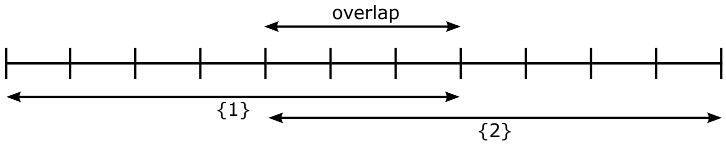

Figure S1: The overlapping Domain Decomposition: *the domain is separated into continuous overlapping parts. Each computational unit computes the corresponding local solution, however, the in-formation in overlap have to be communicated to satisfy the continuity of global solution through domains.*

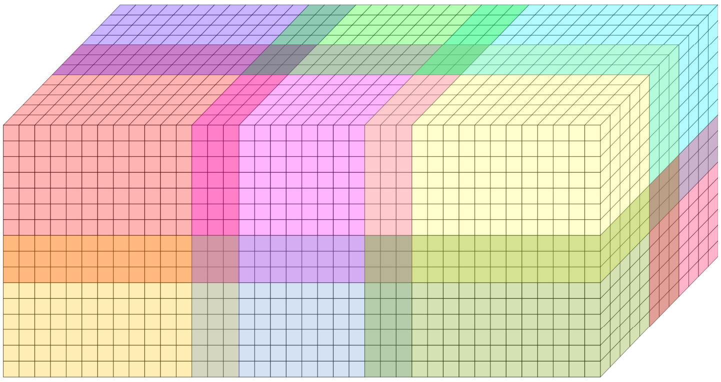

Figure S2: The overlapping Domain Decomposition in 3D: *the simplest way how to decompose the 3D domain into domains is to introduce overlapping rectangular cuboids. Such a decomposition simlifies the implementation and follows the sparsity of Hessian matrix in* Γ *problem.*

suppose that the local unknown part of the solution is $\gamma_{\{d\}}$ and the rest of the solution is $\gamma_{\{.\}}$. Then the objective function can be decomposed into (using the appropriate permutation of indexes)

$$\begin{aligned} f(\gamma_{\{d\}}, \gamma_{\{.\}}) &= \tfrac{1}{2}\gamma_{\{d\}}^T A_{\{d,d\}}\gamma_{\{d\}} - w_{\{d\}}^T\gamma_{\{d\}} + f_{\{.\}}(\gamma_{\{.\}}) + \gamma_{\{d\}}^T A_{\{d,.\}}\gamma_{\{.\}} \\ &= \tfrac{1}{2}\gamma_{\{d\}}^T A_{\{d,d\}}\gamma_{\{d\}} - (w_{\{d\}} - A_{\{d,.\}}\gamma_{\{.\}})^T\gamma_{\{d\}} + f_{\{.\}}(\gamma_{\{.\}}). \end{aligned}$$

In the local domain, the unknown of the problem is the local $\gamma_{\{d\}}$, therefore the term $f_{\{.\}}(\gamma_{\{.\}})$ is constant, does not have any impact on the optimizer, and can be ignored. Please notice that the coupling matrix $A_{\{d,.\}}$ is a block of a sparse matrix and if the size of the overlap is sufficiently larger than the radius of the indicator function of the voxel neighborhood α_0, then the overlap information from the neighboring domains is sufficient information for assembling the correct overall objective function. In the iterative process, we update the linear term using the information from neighbours, solve a new QP problem, and communicate the update of overlap to neighbours, see Algorithm S2. In each iteration, we compare the local solution in overlap with the solution obtained from neighbours and if the difference is sufficiently small, we stop the algorithm.

References

[1] J. Barzilai and J. M. Borwein. Two point step size gradient methods. *IMA Journal of Numerical Analysis*, 8:141–148, 1988.

[2] E. G. Birgin, J. M. Martínez, and M. M. Raydan. Nonmonotone spectral projected gradient methods on convex sets. *SIAM Journal on Optimization*, 10:1196–1211, 2000.

[3] S. Boyd and L. Vandenberghe. *Convex Optimization.* Cambridge University Press, New York, 1st edition, 2004.

[4] Y. Chen and X. Ye. Projection onto a simplex. *Unpublished manuscript, arXiv:1101.6081*, 2011.

[5] L. Grippo, F. Lampariello, and S. Lucidi. A nonmonotone line search technique for Newton's method. *SIAM Journal on Numerical Analysis*, 23(4):707–716, 1986.

[6] D. Mumford and J. Shah. Optimal approximations by piecewise smooth functions and associated variational problems. *Comm. on Pure and Applied Mathematics*, 42(5):577–685, 1989.

Let A, w be data of optimization problem (15) *and let* $\{1\}, \dots, \{N_D\}$ *denote the domains.*
Let $\forall d : \hat{w}_{\{d\}} = w_{\{d\}}$.

repeat

in parallel: solve the local problem

$$\gamma_d^* = \arg_{\gamma_{\{d\}}} \min_{\gamma_{\{d\}} \in \Omega_\Gamma} \frac{1}{2} \gamma_{\{d\}}^T A_{\{d,d\}} \gamma_{\{d\}} - \hat{w}_{\{d\}}^T \gamma_{\{d\}} \tag{23}$$

communication: send and receive the solution in overlap from neighbouring domains $\gamma_{\{.\}}$
in parallel: update linear term

$$\hat{w}_{\{d\}} = w_{\{d\}} - A_{\{d,.\}} \gamma_{\{.\}} \tag{24}$$

until $\|\gamma_{\{d\},overlap} - \gamma_{\{.\}}\|$ *is not sufficiently small*

Return an globally continuous parallel solution $\gamma_{\{1\}}, \dots, \gamma_{\{N_D\}}$.

Algorithm S2: Schwartz Domain Decomposition method for solving Γ-problem in parallel.

[7] L. Pospíšil, P. Gagliardini, W. Sawyer, and I. Horenko. On a scalable nonparametric denoising of time series signals. *Communications in Applied Mathematics and Computational Science*, 13:107–138, 2018.
